# *Toxoplasma* and *Plasmodium* associate with host Arfs during infection

**DOI:** 10.1128/msphere.00770-23

**Published:** 2024-02-13

**Authors:** Erin A. Schroeder, Maria Toro-Moreno, Rene Raphemot, Kayla Sylvester, Isabel C. Colón, Emily R. Derbyshire

**Affiliations:** 1 Department of Molecular Genetics and Microbiology, Duke University School of Medicine, Durham, North Carolina, USA; 2 Department of Chemistry, Duke University, Durham, North Carolina, USA; University at Buffalo, New York, USA

**Keywords:** *Toxoplasma*, *Plasmodium*, Arf, host-parasite interactions, vesicular trafficking

## Abstract

The apicomplexans *Toxoplasma gondii* and *Plasmodium* are intracellular parasites that reside within a host-derived compartment termed the parasitophorous vacuole (PV). During infection, the parasites must acquire critical host resources and transport them across their PV for development. However, the mechanism by which host resources are trafficked to and across the PV remains uncertain. Here, we investigated host ADP ribosylation factors (Arfs), a class of proteins involved in vesicular trafficking that may be exploited by *T. gondii* and *Plasmodium berghei* for nutrient acquisition. Using overexpressed Arf proteins coupled with immunofluorescence microscopy, we found that all Arfs were internalized into the *T. gondii* PV, with most vacuoles containing at least one punctum of Arf protein by the end of the lytic cycle. We further characterized Arf1, the most abundant Arf inside the *T. gondii* PV, and observed that active recycling between its GDP/GTP-bound state influenced Arf1 internalization independent of host guanine nucleotide exchange factors (GEFs). In addition, Arf1 colocalized with vesicle coat complexes and exogenous sphingolipids, suggesting a role in nutrient acquisition. While Arf1 and Arf4 were not observed inside the PV during *P. berghei* infection, our gene depletion studies showed that liver stage development and survival depended on the expression of Arf4 and the host GEF, GBF1. Collectively, these observations indicate that apicomplexans use distinct mechanisms to subvert the host vesicular trafficking network and efficiently replicate. The findings also pave the way for future studies to identify parasite proteins critical to host vesicle recruitment and the components of vesicle cargo.

**IMPORTANCE:**

The parasites *Toxoplasma gondii* and *Plasmodium* live complex intracellular lifestyles where they must acquire essential host nutrients while avoiding recognition. Although previous work has sought to identify the specific nutrients scavenged by apicomplexans, the mechanisms by which host materials are transported to and across the parasite vacuole membrane are largely unknown. Here, we examined members of the host vesicular trafficking network to identify specific pathways subverted by *T. gondii* and *Plasmodium berghei*. Our results indicate that *T. gondii* selectively internalizes host Arfs, a class of proteins involved in intracellular trafficking. For *P. berghei*, host Arfs were restricted by the parasite’s vacuole membrane, but proteins involved in vesicular trafficking were identified as essential for liver stage development. A greater exploration into how and why apicomplexans subvert host vesicular trafficking could help identify targets for host-directed therapeutics.

## INTRODUCTION

Apicomplexans are a diverse class of single-celled eukaryotic parasites that infect nearly all organisms on Earth. Two apicomplexan parasites of significant global health importance are *Toxoplasma gondii* and *Plasmodium* species, which are responsible for toxoplasmosis and malaria, respectively. As obligate intracellular pathogens, they have evolved closely with their hosts to promote replication and evade immune recognition. During invasion, the parasites isolate a portion of the host cell membrane to form an enclosure termed the parasitophorous vacuole (PV). While the parasitophorous vacuole membrane (PVM) is a physical and semipermeable barrier that protects the parasites, it also restricts the free exchange of nutrients and waste with the host cytosol (1, 2). Both *T. gondii* and *Plasmodium* depend on nutrient transport across the PVM to acquire host amino acids, sterols, fatty acids, and other essential nutrients (3, 4). To date, how apicomplexans recruit and transport host resources into the PV remains a critical area of study.

Previous research has uncovered how *T. gondii* and *Plasmodium* reorganize host structures during their intracellular lifecycles. Specifically, both parasites reside at the juxtanuclear position, recruit the microtubule-organizing center, and become enveloped by microtubules that traffic the endoplasmic reticulum (ER), Golgi, and lysosomes to the PV (5–16). *T. gondii* then internalizes select host organelles through deep invaginations of the PVM formed by host microtubules or through fusion with the intravacuolar network (15). Conversely, liver stage *Plasmodium* parasites do not internalize organelles but have been shown to acquire exogenous particles into the PV lumen through an unknown phagocytic mechanism (13). Taken together, these observations suggest that apicomplexans may associate with host organelles to acquire nutrients. Furthermore, the reorganization of host organelles could allow the parasites to interact with the vesicular trafficking network.

The human genome encodes five ADP ribosylation factors (Arfs) that regulate host vesicle trafficking across the endomembrane system (17). Arf proteins (Arf1, Arf3, Arf4, Arf5, and Arf6) are small GTPases responsible for recruiting vesicle coat complexes and modifying membrane lipids. These proteins have different subcellular localizations; Arf6 primarily localizes to the plasma membrane but Arf1, Arf3, Arf4, and Arf5 are found at the Golgi, endosomes, ER, and ER-Golgi intermediate compartment for trafficking. Arf-associated transport between the cis-Golgi and the ER uses coatomer coat complexes (COPI and COPII), while trafficking between the trans-Golgi network and endosomes uses Golgi localized, γ-ear-containing, Arf-binding proteins 1–3 (GGA1-3) (18). Arfs are found in the cytosol and loosely associated with membranes as a GDP-bound inactive monomer. Upon GTP exchange, Arfs integrate into organelle membranes and recruit effector proteins including coat complexes. GTP exchange is facilitated by guanine nucleotide exchange factors (GEFs) while GTP hydrolysis is supported by GTPase-activating proteins (18). Pathogens including *Legionella pneumophila, Chlamydia trachomatis,* and *Salmonella enterica* all subvert host Arfs but their function during apicomplexan infection remains largely unknown (19–21). Apicomplexan parasites are known to fragment and then recruit host Golgi stacks, a process correlated with their viability (6, 11, 12, 22). Thus, Arf proteins, which have a prominent role in Golgi trafficking, are a particularly enticing host protein family to study for their possible role during parasite infection.

In this study, we interrogated host trafficking proteins during *T. gondii* and *Plasmodium berghei* infection of nucleated cells. We found that all human Arf proteins were trafficked to the *T. gondii* PV during early infection and internalized by the end of the lytic cycle. We further characterized host Arf1 as it was the most abundant in the *T. gondii* PV. We found *T. gondii* preferentially internalized Arf1 during active Arf recycling and that the puncta of Arf1 inside the PV colocalized with vesicle coat complexes and exogenous sphingolipids. By contrast, *P*. *berghei* did not associate with Arf1 during the infection of hepatocytes indicating it is not recruited by all apicomplexans. Instead, *P. berghei* development was reduced upon gene depletion of Arf4 and the host GEF, GBF1. Arf4 and GBF1 were also shown to associate with the PVM, highlighting their critical function during the liver stage. Together, this work provides insights into host trafficking machinery hijacked during apicomplexan parasite infection and highlights distinctions between *Toxoplasma* and *Plasmodium*. Future studies exploring the possible function of this pathway in nutrient acquisition and intracellular survival could advance our understanding of critical host-parasite interactions in these deadly pathogens.

## RESULTS

### Host Arfs associate with the *T. gondii PV* throughout the lytic cycle

**Fig 1:**
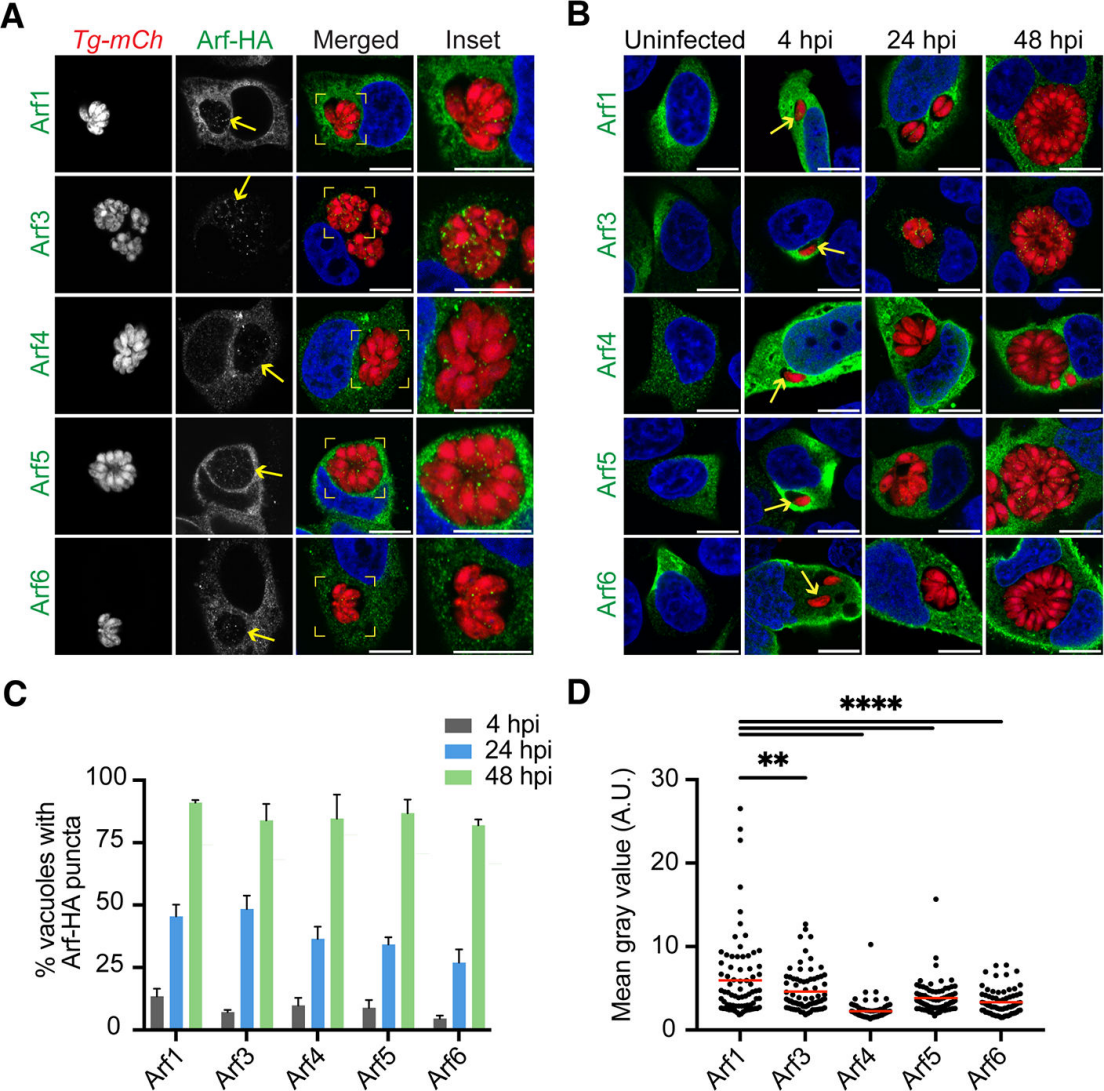
Internalization of host Arfs into the *T. gondii* PV. Representative confocal immunofluorescence images of *Tg-mCh* (red)-infected HeLa cells overexpressing human Arf-HA fusion proteins. Cells were stained with an anti-HA antibody (green) and nuclei were stained with Hoechst (blue). (**A**) At 32 hpi, Arf1, Arf3, Arf4, Arf5, and Arf6 were observed within the *T. gondii* vacuole. Yellow arrows indicate the *T. gondii* PV. Insets (yellow boxes) of the *T. gondii* PV are shown to magnify Arf puncta. (**B**) Arf1, Arf3, Arf4, Arf5, and Arf6 were visualized in uninfected and *Tg-mCh*-infected HeLa cells at 4, 24, and 48 hpi. The enrichment of host Arfs at the PV was observed at 4 hpi (yellow arrows) which were internalized by the end of the lytic cycle at 48 hpi. (**C**) Quantification of *T. gondii* PVs with at least one punctum of host protein at 4 (gray), 24 (blue), and 48 (green) hpi. Data represent mean ± SEM; *n* = 3 biological replicates analyzing >50 PVs per each condition. (**D**) Plot showing the mean Arf fluorescent intensity values within the PV of infected cells at 48 hpi. *n* = 3 biological replicates analyzing a minimum of 20 PVs per replicate. *P*-values display one-way ANOVA with Dunnett’s multiple comparison test for each condition compared to Arf1. **P < 0.01; ****P < 0.0001. Scale bars are 10 µm.

We investigated the localization of host Arfs during *T. gondii* infection using immunofluorescence microscopy. To avoid the potential for antibody cross-reactivity with *T. gondii* Arf1 (Fig. S1), we transfected HeLa cells for 24 hours with plasmids encoding human Arfs (Arf1, Arf3, Arf4, Arf5, and Arf6) fused to hemagglutinin (HA). Twenty-four hours post-transfection, cells were infected with an RH-88 strain of *T. gondii* expressing mCherry (*Tg-mCh).* Infection proceeded for 32 hours, allowing the parasites to undergo at least five rounds of replication before fixation. As a control, HeLa cells were transfected with a plasmid encoding GFP, infected with *Tg-mCh*, and fixed at 32- and 48 hours post-infection (hpi). Uninfected and *Tg-mCh*-infected cells were stained with Hoechst before visualization by confocal microscopy. We observed no internalization of GFP into the *T. gondii* vacuole at either time point (Fig. S2A). On the other hand, we observed puncta of Arf1, Arf3, Arf4, Arf5, and Arf6 inside the PV that is associated with *T. gondii* at 32 hpi (Fig. 1A).

To further understand host Arf internalization during infection, we quantified the percentage of PVs containing at least one punctum of host Arfs at various times post-infection. We examined Arf recruitment during invasion (0.5 hpi), after invasion but before the first replication cycle (4 hpi), halfway through the lytic cycle (24 hpi), and during late lytic development when parasites prepare for egress (48 hpi). During parasite invasion (0.5 hpi), we observed a gathering of Arf6 at the PVM. This phenotype was unsurprisingly unique to Arf6 given that it is the only Arf to localize at the host cell membrane for trafficking (Fig. S2B). All Arfs then followed the same pattern of localization during the lytic cycle. Before parasite replication (4 hpi), less than 20% of *T. gondii* PVs had enrichment of Arfs at the host-parasite interface (Fig. 1B and C; Fig. S2). As *Tg-mCh* replicated (24 hpi), intense speckles of Arfs were seen in uneven patterns between the individual parasites in about 50% of all vacuoles. By the end of the lytic cycle (48 hpi), each Arf was found in more than 80% of *T. gondii* PVs (Fig. 1C). Thus, as infection progressed (4–48 hpi), more *T. gondii* PVs internalized host Arfs, indicating that these host proteins could be important during the later stages of the lytic cycle. These results are consistent with previous studies that described Arf6 during *T. gondii* infection. Specifically, Vieira da Silva *et al*. found an accumulation of Arf6 during the invasion, and Hartman *et al*. reported the internalization of Arf6 into the *T. gondii* PV at 24 hpi (23, 24).

To quantify Arf recruitment, we completed an analysis of the mean gray value of each Arf within the *T. gondii* PV as previously implemented by Romano *et al*. with the *T. gondii* PV (6, 25). While all Arfs are internalized*,* we found Arf1 was the most abundant in the *T. gondii* PV (Fig. 1D). Importantly, we observed no linear relationship between the host expression of Arf1 and the internalization of Arf1 into the PV, suggesting that higher Arf expression did not influence our abundance analysis (Fig. S3). To focus our efforts on Arf1, we asked whether endogenous *Arf1* expression levels changed during *Tg-mCh* infection. Previously published RNA-seq data sets have reported no significant changes in *Arf1* expression levels in human foreskin fibroblasts and mouse models of acute and chronic *T. gondii* infection (Table S1) (26, 27). To confirm, we used qRT-PCR and assessed *Arf1* expression levels at 4, 24, and 48 hpi of *Tg-mCh* in HeLa cells (Fig. S4). Consistent with previous findings, we observed no significant change in *Arf1* expression, suggesting that while *T. gondii* can sequester Arf1 into its PV, it does not alter its expression.

### Proper recycling of Arf1 is important for its internalization into the *T. gondii* PV

Arf recycling from a GDP-bound to a GTP-bound state is essential for cargo sorting and vesicle formation (18). We investigated whether active Arf1 recycling was a prerequisite for its internalization using the well-characterized point mutations in the Arf-GTP binding pocket that creates a constitutively active (Arf1-Q71L) or dominant negative (Arf1-T31N) protein state (28). Since Arf1-Q71L is constitutively bound to GTP, the protein still promotes vesicle formation and budding but the vesicles cannot dock to their target membranes. As a result, Arf1-Q71L remains bound to vesicle and organelle membranes. By contrast, Arf1-T31N cannot bind GTP so it fails to associate with membranes and redistributes in the cytosol (28).

**Fig 2:**
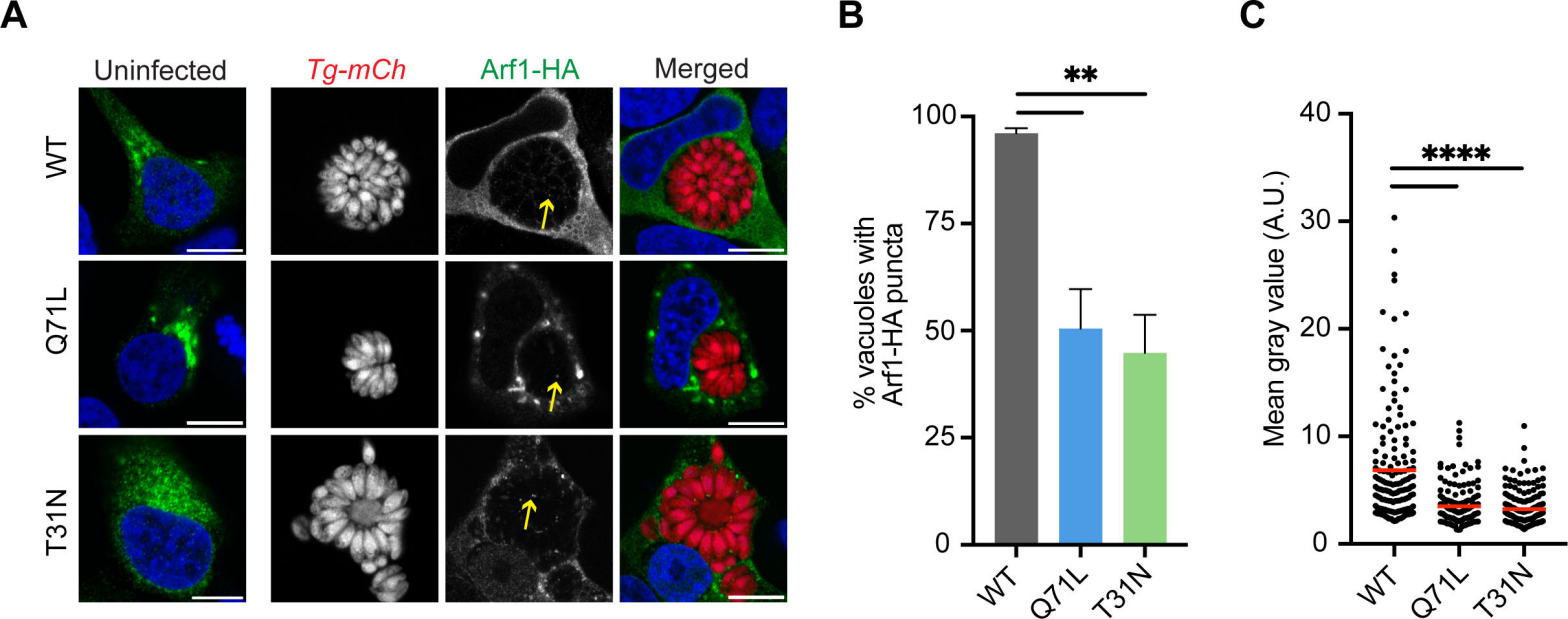
Active Arf1 recycling is essential for its complete internalization. (**A**) Representative confocal immunofluorescence images of uninfected and *Tg-mCh* (red)-infected HeLa cells overexpressing Arf1-WT, the constitutively active GTP-bound Arf1-Q71L, and the dominant-negative GDP-bound Arf1-T31N at 48 hpi. Cells were stained with anti-HA (green) and nuclei were stained with Hoechst (blue). Yellow arrows indicate Arf1-HA puncta inside the PV. Scale bars are 10 µm. (**B**) Quantification of *T. gondii* PVs with at least one punctum of Arf1-WT (gray), Arf1-Q71L (blue), or Arf1-T31N (green) at 48 hpi. Data represent mean ± SEM, *n* = 3 biological replicates analyzing >50 PVs for each condition. *P*-values display one-way ANOVA with Dunnett’s multiple comparison test for each condition compared to Arf1-WT. **P < 0.01. (**C**) Plot showing the mean fluorescent intensity values for Arf1-WT, Arf1-Q71L, and Arf1-T31N within the PV of *Tg-mCh-*infected HeLa cells at 48 hpi. *n* = 3 biological replicates analyzing >50 PVs for each condition. *P*-values display one-way ANOVA with Dunnett’s multiple comparison test for each condition compared to Arf1-WT. ****P < 0.0001.

HeLa cells were transfected with Arf1-WT, Arf1-Q71L, and Arf1-T31N as HA fusion proteins before infection with *Tg-mCh.* At 48 hpi, the cells were fixed, stained with anti-HA, and visualized by microscopy (Fig. 2A; Fig. S5A). In uninfected HeLa cells, we observed the characteristic phenotypes of the Arf1-Q71L and Arf1-T31N mutants. Arf1-Q71L formed aggregates within the cytosol as it failed to disassociate with membranes while Arf1-T31N became dispersed. In *Tg-mCh-*infected cells, the number of *T. gondii* vacuoles that internalized Arf1 significantly decreased by approximately 50% in cells overexpressing either the constitutively active or dominant negative Arf1 protein (Fig. 2B). We also found vacuoles that internalized either mutant Arf1-Q71L or Arf1-T31N contained only a few puncta, whereas Arf1-WT was observed in a greater abundance. We calculated the mean gray value for the Arf1 signal inside the PV and found a significant decrease in the amount of Arf1 trafficked inside the vacuole for both mutant proteins (Fig. 2C). These findings indicate that without proper Arf1 recycling, internalization into the *T. gondii* PV is reduced. Moreover, we found a significant reduction in PV size at 48 hpi for cells overexpressing either Arf1-Q71L or Arf1-T31N when compared to Arf1-WT (Fig. S5B).

### Arf1 is trafficked to the *T. gondii* PV independent of host GEF activity

**Fig 3:**
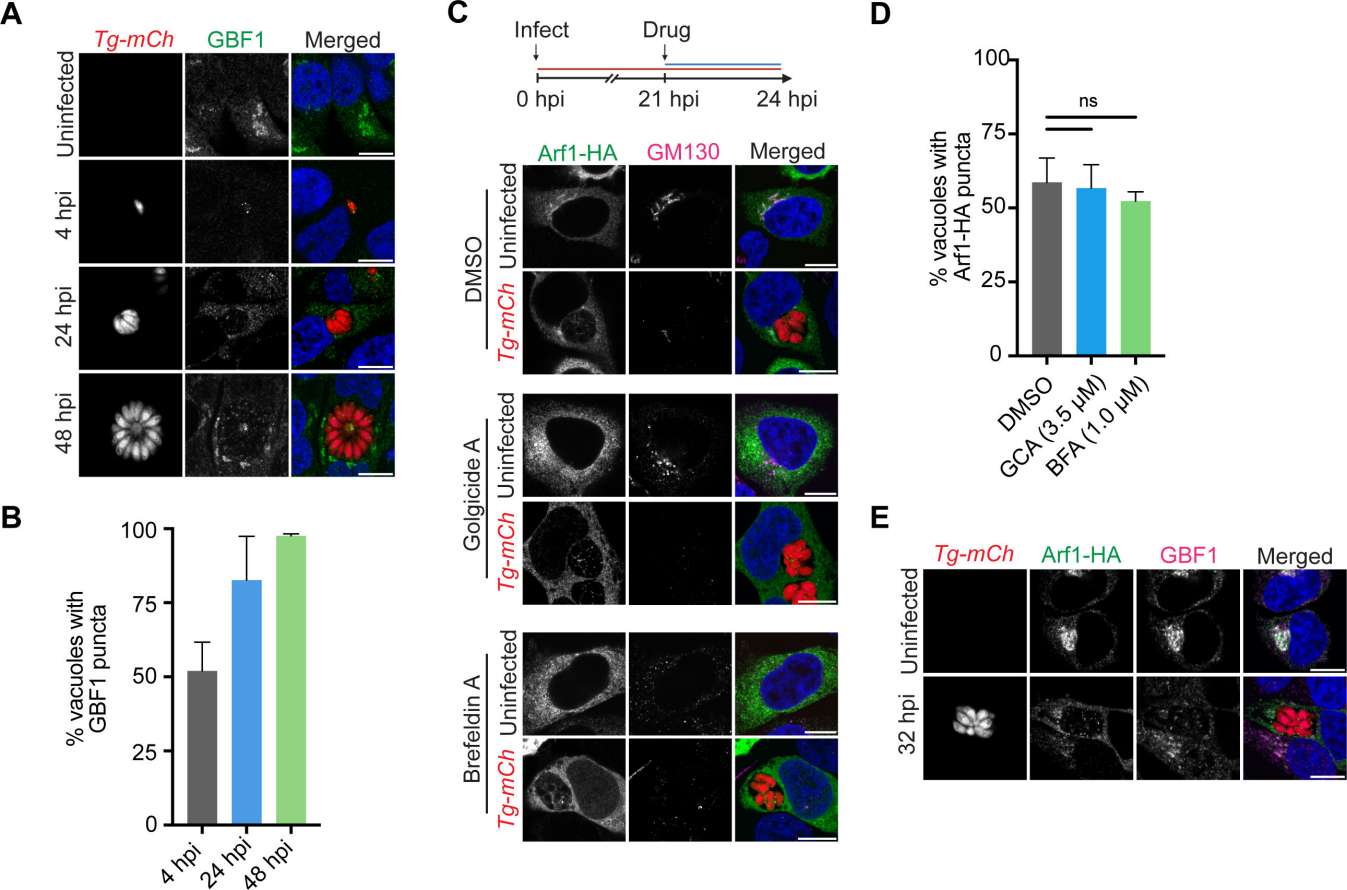
Arf1 and GBF1 are uncoupled in the *T. gondii* PV. (**A**) Representative confocal immunofluorescence images of uninfected and *Tg-mCh* (red)-infected HeLa cells at 4, 24, and 48 hpi. Cells were stained with anti-GBF1 (green) and nuclei were stained with Hoechst (blue). (**B**) Quantification of *T. gondii* PVs with at least one punctum of GBF1 at 4 (gray), 24 (blue), and 48 (green) hpi. Data represent mean ± SEM, *n* = 3 biological replicates analyzing >50 PVs for each condition. (**C**) Assay schematic showing drug administration at 21 hpi and analysis at 24 hpi. Representative confocal immunofluorescence images of uninfected and *Tg-mCh* (red)-infected HeLa cells overexpressing Arf1-HA and treated with DMSO, 3.5 µM golgicide A (GCA), or 1.0 µM brefeldin A (BFA). Cells were stained with anti-HA (green), anti-GM130 (magenta), and nuclei were stained with Hoechst (blue). (**D**) Quantification of *T. gondii* PVs with at least one punctum of Arf1 at 24 hpi after treatment with DMSO (gray), GCA (blue), or BFA (green). Data represent the percentage of *T. gondii* PVs with at least one punctum of Arf1 compared to the DMSO control ±SEM, *n* = 3 biological replicates analyzing >50 PVs for each condition. *P*-values display one-way ANOVA with Dunnett’s multiple comparison test for each condition compared to DMSO. ns = non-significant. (**E**) Representative confocal immunofluorescence images of uninfected and *Tg-mCh* (red)-infected HeLa cells overexpressing Arf1-HA at 32 hpi. Cells were stained with anti-HA (green), anti-GBF1 (magenta), and nuclei were stained with Hoechst (blue). Scale bars are 10 µm.

Arf recycling and GTP exchange are facilitated by a family of host GEF proteins (18). We hypothesized that if Arf1 recycling was important for its internalization, a specific host GEF may support the association of Arf1 to the *T. gondii* PV. To test this, we focused on GBF1, the canonical Arf1 GEF that regulates vesicle trafficking from the Golgi to the ER (29). We began by characterizing the location of endogenous GBF1 during the *T. gondii* lytic cycle. Uninfected and *Tg-mCh*-infected HeLa cells were stained with an anti-GBF1 antibody at 4, 24, and 48 hpi. GBF1 was internalized by the *T. gondii* PV starting early in infection (4 hpi) with nearly 100% of *T. gondii* vacuoles containing at least one punctum by the end of the lytic cycle (48 hpi) (Fig. 3A and B). To confirm this recruitment, YFP-GBF1 was overexpressed in *Tg-mCh-*infected HeLa cells and visualized (30). We found YFP-GBF1 was also internalized into the *T. gondii* PV (Fig. S6).

To further explore the role of GBF1 during infection, we employed the well-characterized chemical inhibitor golgicide A (GCA) to selectively decrease GBF1-mediated Arf1 activation (31). We treated uninfected and *Tg-mCh*-infected HeLa cells overexpressing Arf1-HA with 3.5 µM GCA for 3 hours before fixation at 24 hpi (Fig. 3C). Cells were stained with GM130 to mark the cis-Golgi and anti-HA to visualize Arf1. In uninfected control cells, GCA caused the host cis-Golgi to disassemble and Arf1 to be released from the Golgi membrane, as expected (Fig. 3C) (31). In *Tg-mCh-*infected cells, we observed Golgi disruption upon GCA treatment, but parasites remained capable of Arf1 internalization. To distinguish differences in Arf1 internalization during GCA treatment, the number of *T. gondii* vacuoles that contained at least one punctum of Arf1 was quantified. The percentage of vacuoles that internalized Arf1 remained unchanged between DMSO and GCA treatment, suggesting that GBF1 was not required for Arf1 trafficking to the PV (Fig. 3D). Moreover, while GBF1 and Arf1 colocalized at the host Golgi, they did not colocalize in the *T. gondii* PV. In the parasite vacuole at 32 hpi, Arf1 existed as puncta of protein around the tachyzoites, while GBF1 appeared to associate with the parasite’s apical region that houses its Golgi complex (Fig. 3E). Thus, while GBF1 might play a role during infection, its function appears to be independent of Arf1.

Arf1 also interacts with several other GEFs (i.e., BIG1 and BIG2) at the trans-Golgi network for protein sorting and endosome trafficking (18). We investigated whether these additional GEF proteins could support Arf1 recycling and internalization by employing brefeldin A (BFA), a small molecule inhibitor that indiscriminately targets the Sec7 domain of GEF proteins (31). Uninfected and *Tg-mCh*-infected HeLa cells overexpressing Arf1-HA were treated for 3 hours with 1 µM BFA before fixation at 24 hpi (Fig. 3C). As expected, BFA treatment caused the complete disassembly of the host cis-Golgi in uninfected cells; however, no significant change in the number of *T. gondii* vacuoles that internalized Arf1 was observed between DMSO and BFA treatment (Fig. 3C and D). To determine whether host GEF activity would regulate Arf1 internalization later in infection, we treated *Tg-mCh*-infected cells with DMSO, GCA, or BFA from 29 to 32 hpi. Again, we observed no significant difference in the number of vacuoles that internalized Arf1 with inhibitor treatment (Fig. S7A and B). Together, these results suggest that neither the host Golgi nor GEF function is required for Arf1 internalization by the *T. gondii* PV.

### Arf1 colocalizes with host vesicle coat complexes and sphingolipids in the *T. gondii* PV

**Fig 4:**
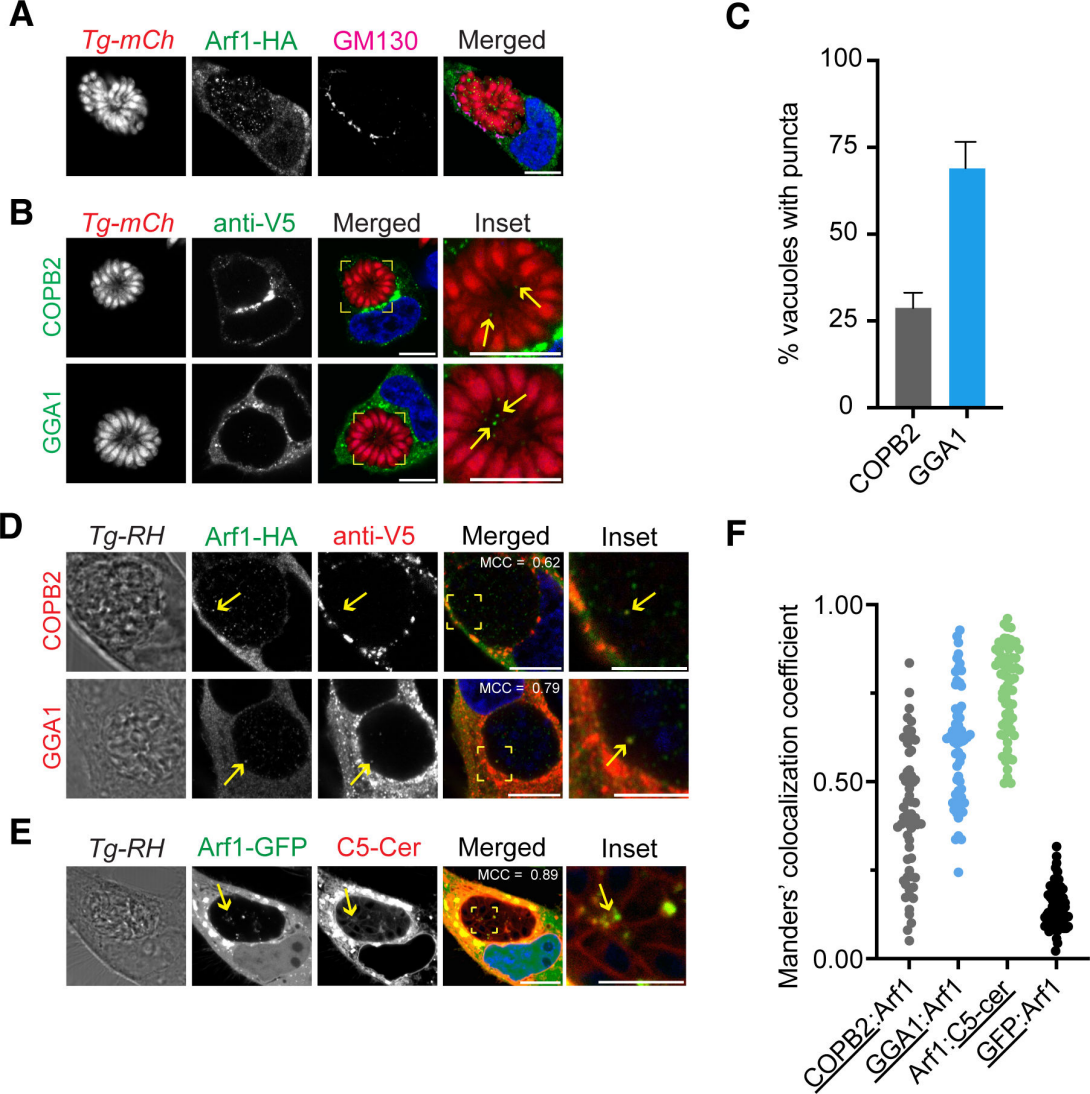
Arf1 colocalizes with COPB2 and GGA1 in the *T. gondii* PV. (**A**) Representative confocal immunofluorescence images of the host cis-Golgi (GM130) in *Tg-mCh* (red)-infected HeLa cells overexpressing Arf1-HA at 48 hpi. Cells were stained with anti-HA (green), anti-GM130 (magenta), and nuclei were stained with Hoechst (blue). (**B**) Representative confocal immunofluorescence images of *Tg-mCh* (red)-infected HeLa cells overexpressing COPB2-V5 and GGA1-V5 at 48 hpi. Cells were stained with anti-V5 (green) and nuclei were stained with Hoechst (blue). Insets (yellow boxes) of the *T. gondii* PV are shown to magnify the puncta (yellow arrows) of each vesicle coat complex inside of the *T. gondii* PV. (**C**) Quantification of *T. gondii* PVs with at least one punctum of COPB2 and GGA1 at 48 hpi. Data represent mean ± SEM, *n* = 3 biological replicates analyzing >50 PVs for each condition. (**D**) Representative confocal immunofluorescence images of untagged *T. gondii*-infected HeLa cells overexpressing both Arf1-HA and COPB2-V5 or GGA1-V5 at 48 hpi. Cells were stained with anti-HA (green), anti-V5 (red), and nuclei were stained with Hoechst (blue). Insets (yellow boxes) are shown to magnify the colocalization of Arfs and coat complexes within the *T. gondii* PV (yellow arrows). (**E**) Representative confocal immunofluorescent images of untagged *T. gondii-*infected HeLa cells overexpressing Arf1-GFP (green) at 48 hpi. Cells were treated with 5 µM BODIPY-TR C5-ceramide (red) from 24 to 48 hpi. Yellow arrows indicate puncta of Arf1-GFP and BODIPY-TR C5-ceramide that colocalize within the *T. gondii* PV. An inset (yellow boxes) is shown to magnify the colocalization of Arf1 and BODIPY-TR C5-ceramide. (**F**) The Manders’ colocalization coefficient (MCC) of Arf1 with COPB2, GGA1, and BODIPY-TR C5-ceramide was determined for *T. gondii* PVs. Data represent COPB2 to Arf1, GGA1 to Arf1, and Arf1 to C5-cer. As a negative control, the MCC of GFP to Arf1 was determined inside of the *T. gondii* PV for cells overexpressing both proteins. *n* = 3 biological replicates analyzing >20 PVs for each condition. Scale bars are 10 µm, except for 4D and 4E insets, which are 5 µm.

To further interrogate Arf1 internalization by *T. gondii*, we performed colocalization studies with different host membrane markers. Arf1 primarily localizes to the host Golgi, an organelle that is well characterized to undergo fragmentation during *T. gondii* infection (6). To determine whether the punctum of host Arf1 observed inside the PV represented Golgi fragments, we performed co-staining for the cis-Golgi marker GM130 in HeLa cells overexpressing Arf1-HA (Fig. 4A). As previously reported, we observed the host cis-Golgi fragmented around the *T. gondii* PV (6). However, no Golgi fragments colocalized with Arf1-HA inside the *T. gondii* PV, indicating the Arf1 puncta did not stem from internalized Golgi fragments.

Alternatively, *T. gondii* could internalize Arf1 vesicles into its PV. Arf1 mobilizes COPI at the cis-Golgi for trafficking to the ER and recruits GGA1 for trafficking from the trans-Golgi (29). The COPI protein complex is composed of seven subunits in mammalian cells with COPI coat complex subunit beta 2 (COPB2) commonly used to represent the localization of the full complex. Given that *T. gondii* has homologs for multiple COPI subunits, we performed overexpression studies of COPB2 and GGA1 fused to a V5-tag to avoid the potential for antibody cross-reactivity (32). At 48 hpi, we observed puncta of COPB2 and GGA1 inside the *T. gondii* PV near the residual body (Fig. 4B). We found that approximately 30% of vacuoles contained COPB2 puncta while GGA1 was observed in about 70% of vacuoles at 48 hpi (Fig. 4C). We then overexpressed both Arf1-HA with COPB2-V5 or GGA1-V5 and infected HeLa cells with an untagged RH-88 strain of *T. gondii*. To assess colocalization, we quantified the Manders’ colocalization coefficient (MCC) of COPB2 and GGA1 with Arf1 within the *T. gondii* PV for at least 20 PVs per biological replicate. The MCC determines the percentage of the total signal from one channel that overlaps with the signal from another such that an MCC of 0.5 indicates a 50% overlap between the two signals. Based on this analysis, a moderate colocalization of the COPB2 or GGA1 puncta to Arf1 puncta was observed (Fig. 4D and F). As a negative control, we overexpressed GFP and Arf1-HA in HeLa cells infected with untagged RH-88 strain of *T. gondii* and determined the MCC within the *T. gondii* PV (Fig. 4F; Fig. S8). This analysis indicated no colocalization (MCC <0.5) was observed with GFP and Arf1-HA, as expected.

Given the known dependence of *T. gondii* on host sphingolipids, we hypothesized the parasites may use Arf1 to recruit lipids (6, 15, 33, 34). We infected HeLa cells overexpressing Arf1-GFP with an untagged RH-88 strain of *T. gondii*. Cells were then treated with BODIPY TR C5-ceramide from 24 to 48 hpi before live cell microscopy. We found that BODIPY TR C5-ceramide was successfully trafficked inside the PV at 48 hpi. Furthermore, we found that Arf1-GFP puncta colocalized with BODIPY TR C5-ceramide signal within the PV (Fig. 4E). An analysis of the MCC for at least 20 PVs per biological replicate revealed a strong relationship between Arf1-GFP and BODIPY TR C5-ceramide (Fig. 4F). These findings indicate that Arf1 may facilitate the trafficking of host sphingolipids into the PV.

### Arf4 and GBF1 are important for *P. berghei* liver stage infection

We have previously reported the importance of host vesicular trafficking machinery in the related apicomplexan *P. berghei* (35). Specifically, we found that GGA1 and two subunits of COPI vesicles (COPB2 and COPG1) were critical to *P. berghei* liver stage development and that GGA1 and COPB2 were recruited to the PVM of both *P. berghei* and *Plasmodium yoelii* at 48 hpi (35). Furthermore, a recent CRISPR-cas9 screen during the *Plasmodium* liver stage found significant enrichment for gene ontology terms associated with vesicular trafficking (12). Thus, we sought to investigate the function and localization of host Arfs during the *P. berghei* liver stage.

**Fig 5:**
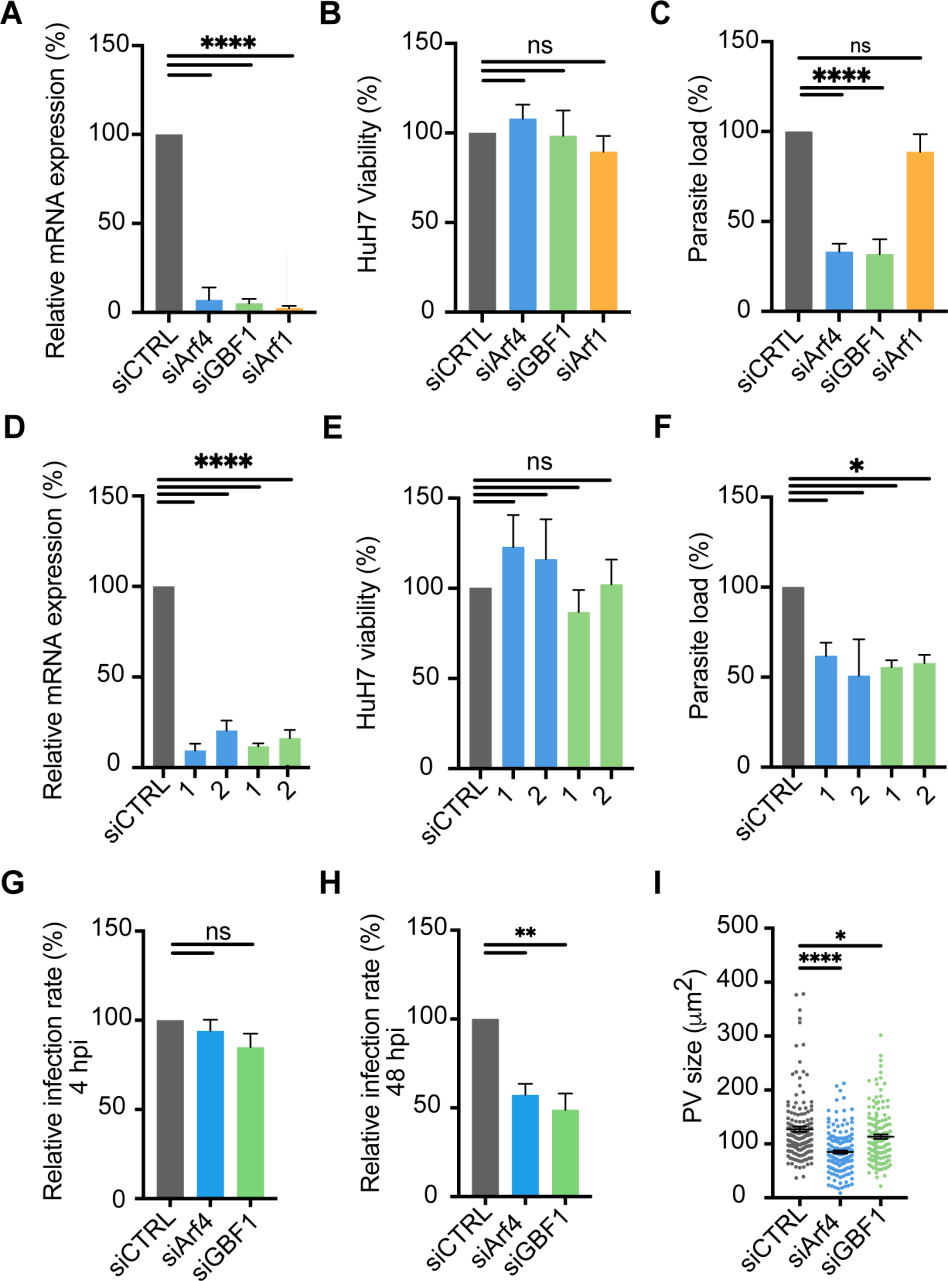
Arf4 and GBF1 depletion impairs *P. berghei* liver stage development. (**A-C**) HuH7 cells were reverse transfected (15 nM) for 48 hours with a non-targeting scramble control (siCTRL, gray) or two pooled siRNAs targeting Arf4 (blue), GBF1 (green), and Arf1 (orange). (**A**) The relative mRNA levels of HuH7 cells treated with pooled siRNAs were determined by qRT-PCR. Samples were normalized to *Hs*18S and compared to cells treated with siCTRL. (**B**) Forty-eight hours post-siRNA transfection, cells were infected with *Pb-*Luc sporozoites. HuH7 cell viability was assessed at 48 hpi using a CellTiter-Fluor assay. Data are normalized to siCTRL. (**C**) The parasite load was assessed at 48 hpi and normalized to cells treated with siCTRL. (**D-F**) HuH7 cells were reverse transfected (15 nM) for 48 hours with a non-targeting scramble control (siCTRL, gray) or two different siRNAs targeting Arf4 (1 and 2, blue) and GBF1 (1 and 2, green). (**D**) The relative mRNA levels of HuH7 cells treated with single siRNAs were determined by qRT-PCR. Samples were normalized to *Hs*18S and compared to cells treated with siCTRL. (**E**) Forty-eight hours post-siRNA transfection, cells were infected with *Pb-*Luc sporozoites. HuH7 cell viability was assessed at 48 hpi using a CellTiter-Fluor assay. Data are normalized to siCTRL. (**F**) The parasite load was assessed at 48 hpi and normalized to cells treated with siCTRL. (**G-I**) Cells were reverse transfected with two pooled siRNAs (15 nM) for a non-targeting scramble control, Arf4, and GBF1. The (**G**) relative infection rate at 4 hpi, (**H**) relative infection rate at 48 hpi, and (**I**) PV size at 48 hpi were assessed for a non-targeting scramble control (siCTRL, gray), Arf4 (blue), and GBF1 (green). Data for the relative infection rates were normalized to cells treated with siCTRL. (**A-I**) Data represent mean ± SEM, *n* = 3 biological replicates. *P*-values display one-way ANOVA with Dunnett’s multiple comparison test for each condition compared to siCTRL. ns = non-significant *P < 0.05; **P < 0.01; ****P < 0.0001.

In addition to Arf1 and GBF1, COPI retrograde vesicles are regulated by Arf4. To interrogate the functional role of these host proteins during the *P. berghei* liver stage infection, we used two pooled siRNAs to knockdown mRNA expression of *Arf1, Arf4,* and *GBF1* in HuH7 cells infected with luciferase-expressing *P. berghei* (*Pb*-Luc). At 48 hpi, we found that siRNA knockdown of *Arf1, Arf4,* and *GBF1* had no significant impact on HuH7 cell viability (Fig. 5A and B). Interestingly, *Arf1* knockdown had no impact on parasite load while *Arf4* and *GBF1* depletion led to a significant reduction in *P. berghei* load (Fig. 5C). We next completed this analysis with single siRNAs against *Arf4* and *GBF1* to confirm the phenotype. After *Arf4* and *GBF1* depletion, we observed a significant decrease in *P. berghei* load in HuH7 cells with two separate siRNAs but no impact on host cell viability (Fig. 5D through F). We then investigated whether *Arf4* and *GBF1* were important for *P. berghei* invasion and/or development. We found gene depletion of *Arf4* and *GBF1* had no impact on the relative infection rate at 4 hpi, indicating that parasite invasion was not hindered (Fig. 5G). However, both the relative infection rate and PV size significantly decreased at 48 hpi with *Arf4* and *GBF1* depletion (Fig. 5H and I). These findings indicate that Arf4 and GBF1 are critical for parasite development and survival during the liver stage.

**Fig 6:**
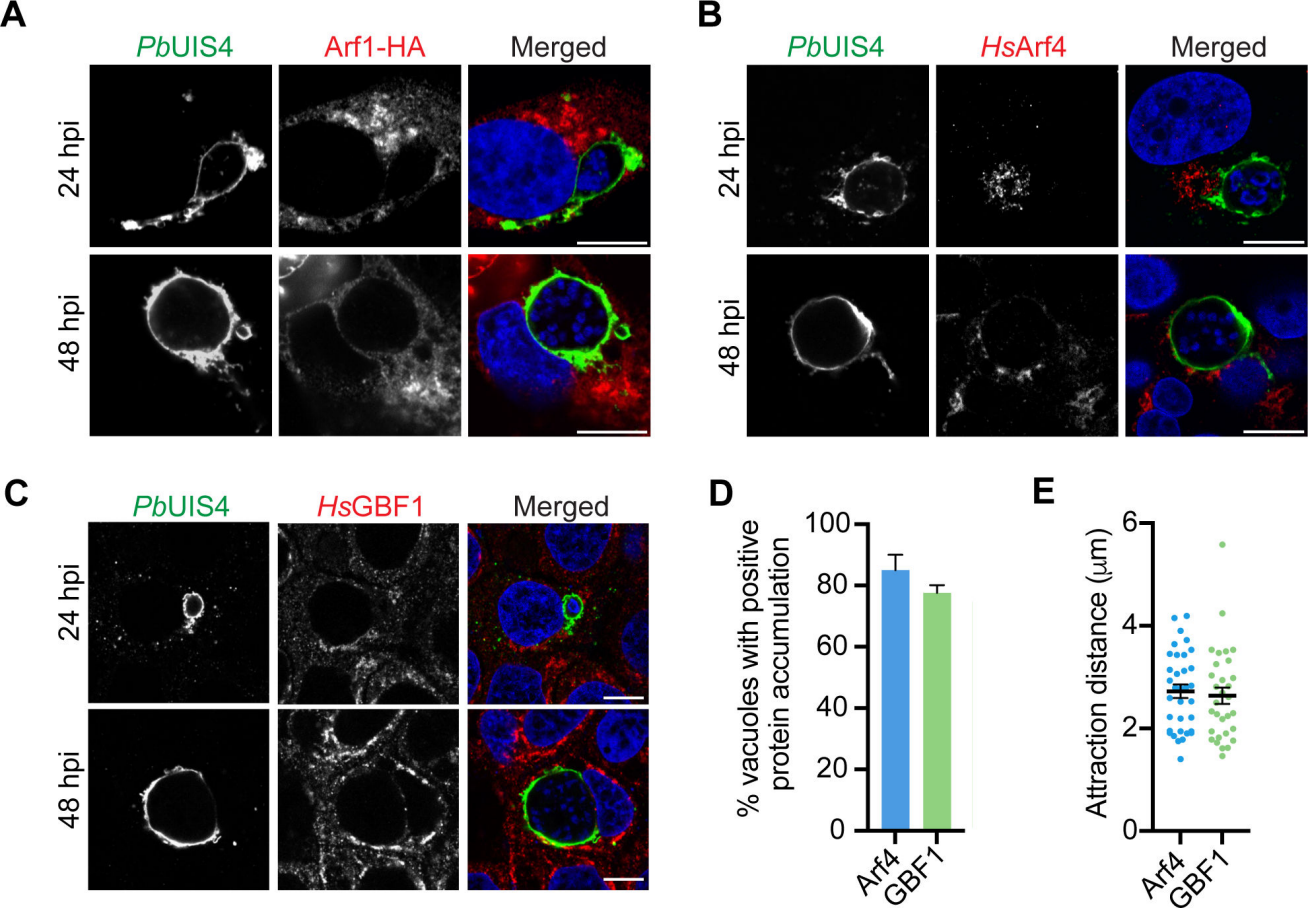
Arf4 and GBF1 are associated with the *P. berghei* PVM. (**A**) Representative confocal immunofluorescence microscopy images of *P. berghei*-infected HeLa cells overexpressing Arf1-HA at 24 and 48 hpi. Arf1-HA did not colocalize with the *P. berghei* PVM resident protein UIS4. Cells were stained with anti-HA (red), anti-USI4 (green), and nuclei were stained with Hoechst (blue). (**B and C**) *P. berghei*-infected HuH7 cells *at* 24 and 48 hpi were stained with (**B**) anti-Arf4 (red) or (**C**) anti-GBF1 (red). Cells were stained with anti-UIS4 (green) and nuclei were stained with Hoechst (blue). Scale bars are 10 µm. (**D and E**) The percentage of *P. berghei* vacuoles with Arf4 or GBF1 protein accumulation was assessed using Z-stacks of *P. berghei*-infected HuH7 cells at 48 hpi with Imaris software. (**D**) Protein accumulation to UIS4 was scored positive if the amount of Arf4 or GBF1 spots at the vacuole was statistically greater than a random distribution of protein within the host cell. (**E**) The attraction distance was calculated for vacuoles with a positive accumulation of Arf4 or GBF1 to the UIS4 surface. The value represents the distance from the UIS4 surface where there was a statistically greater accumulation of Arf4 or GBF1 than a random simulation. Data represent mean ± SEM, *n* = 3 biological replicates analyzing 20 PVs for each condition.

We next studied the localization of Arf1, Arf4, and GBF1 during *P. berghei* liver stage infection. Since we were unable to validate a selective Arf1 antibody, we overexpressed Arf1-HA in HeLa cells and infected them with *Pb*-Luc. Cells were fixed at 24 or 48 hpi to represent mid- and late liver stage development, and stained for anti-HA and the PVM resident protein UIS4 (36). We observed that Arf1-HA was not internalized by the vacuole nor enriched at the *P. berghei* PVM at either 24 or 48 hpi (Fig. 6A). This finding is distinct from our results with *T. gondii*, which recruited all five Arf proteins. We next examined the location of Arf4 and GBF1, both of which were important for *P. berghei* liver stage development. For these experiments, we could examine the location of endogenous protein as we had validated Arf4 and GBF1 antibodies. HuH7 cells were infected with *Pb*-Luc, fixed at 24 or 48 hpi, and subsequently stained with anti-UIS4 and anti-Arf4 or anti-GBF1 (Fig. 6B and C). We observed that both Arf4 and GBF1 were associated with the PVM. Arf4 and GBF1 association resembled fragmented Golgi stacks as recently reported in two independent studies (11, 12). This is distinct from the association previously observed for COPB2 and GGA1 which colocalized uniformly with UIS4 (35).

To further study the accumulation of endogenous Arf4 and GBF1 at the PVM, we obtained Z-stacks of *Pb*-Luc-infected HuH7 cells and compared a random distribution of either protein to their actual localization within the host cell. Positive attraction to the UIS4 surface was assessed when the distribution of Arf4 and GBF1 spots deviated from 98% of the simulations for a random distribution (Fig. 6D). Examples of infected cells without a positive attraction of Arf4 and GBF1 to the UIS4 surface are shown *(*Fig. S9*).* We found that approximately 80% of the cells showed a positive accumulation of Arf4 and GBF1 at the parasite vacuole, and on average, this accumulation was around 2 µm from the PVM surface (Fig. 6E). A similar analysis could not be performed with Arf1 since the protein can only be observed after Arf1-HA overexpression (i.e., not endogenous protein). However, visualization of Arf1-HA overexpression in *Pb*-infected versus *Tg*-infected cells suggests Arf1 is only recruited to the *T. gondii* PV. Furthermore, our gene depletion and protein recruitment studies are consistent, suggesting the observed recruitment of Arf4 and GBF1 may support *Plasmodium* liver stage survival.

## DISCUSSION

*T. gondii* and *P. berghei* remodel host organelles and the microtubule network to establish a protective and nutrient-rich environment [reviewed for *T. gondii* in (37) and *P. berghei* in (4)]. Significant research has focused on characterizing the host Golgi given that making contact with and fragmenting the organelle appears to be critical for parasite development (5, 6, 11, 12). Modifying the host Golgi could serve several purposes: to acquire nutrients, to inhibit the presentation of immune signaling molecules, and/or to gain proximity to host vesicular trafficking. To further interrogate host trafficking in the context of *T. gondii* and *P. berghei* infection, we characterized the location of Arf GTPases. We found that the entire Arf family was trafficked inside the *T. gondii* PV and validated previous reports that Arf6 accumulated around invading parasites and was internalized during parasite development (23, 24). Furthermore, we discovered that Arf1 was recruited in the greatest abundance and active Arf1 recycling independent of host GEF activity promoted internalization into the PV, suggesting a putative parasite protein could regulate Arf1 activation. Interestingly, Arf movement during *T. gondii* infection was distinct from *Plasmodium*, where Arf4 (not Arf1) was recruited to the parasite vacuole but not internalized. Depletion of host *Arf4* and *GBF1* reduced *P. berghei* liver stage development and survival with no impact on HuH7 viability. These host proteins may directly aid in parasite development, or their host-dependent roles are indirectly impacting parasite viability. While *T. gondii* and *P. berghei* interact with host Arfs differently, our findings build on the growing evidence that apicomplexan parasites leverage the host trafficking machinery for survival.

In addition to our study of the Arf family, a second class of small GTPases, Ras-associated binding proteins (Rabs), are also involved in host vesicular trafficking and have been studied in the context of apicomplexans. To date, 15 Rabs have been found inside the *T. gondii* PV (6, 15). Rab11A puncta have been specifically characterized as *bona fide* vesicles inside the PV using electron microscopy. While the precise mechanism for Rab internalization is unknown, it is postulated that microtubules carrying Rab vesicles may form deep pockets in the PVM. Then through a phagocytic-like process, vesicles are internalized and engulfed by the *T. gondii* intravacuolar network for vesicle and cargo degradation (15). For *P. berghei*, there is no evidence that the parasites internalize host Rabs or vesicles. However, Rab1A, Rab11A, and Rab14 are all essential for liver stage development, and Rab7A, which localizes to late endosomes, were found to be enriched around the PVM (13, 38, 39). Moreover, Rab14 and nine other Rabs are upregulated during *P. berghei* infection, indicating that the parasite may modulate host gene expression to increase protein trafficking (40). These findings on Rab GTPases thus complement our observations. Arfs are internalized by *T. gondii* but restricted by the *P. berghei* PVM. Furthermore, like multiple Rabs, *Arf4* was found to promote *P. berghei* survival and development without being recruited inside the PV.

*T. gondii* can differentially internalize specific Rab and Arf GTPases into its PV. Rabs from the recycling, secretory, and anterograde pathways are all preferentially internalized by the parasite and we found that Arf1 was internalized with the greatest frequency when compared to other Arf proteins (6, 15). Likewise, *T. gondii* can internalize host endocytic organelles and multivesicular bodies while excluding peroxisomes and ER elements (8, 15). This bias supports a parasite mechanism that can identify and divert specific host vesicles and organelles to its PV. Identifying the parasite protein(s) that support such a mechanism as well as what resources they seek to sequester will be critical next steps to further understand this host-parasite interaction. In the case of Arf1, Rab14, Rab30, and Rab43, these predicted vesicles colocalize with exogenous BODIPY TR C5-ceramide and may support the trafficking of sphingolipids and perhaps other essential nutrients and proteins into the PV (6).

Arf1 has multiple functions that could explain why it is targeted by *T. gondii*. Canonically, Arf1 is associated with vesicular trafficking at the Golgi (29). We found that COPB2 and GGA1, two components of vesicular coat complexes, were also internalized into the *T. gondii* PV. Importantly, the COPB2 and GGA1 puncta colocalized with Arf1 suggesting that these host proteins could exist as *bona fide* vesicles. We also observed puncta of COPB2 and GGA1 that did not colocalize with Arf1 inside the PV, which was particularly variable with COPB2. It is possible that other members of the Arf family are activating those vesicles, or the observed puncta could represent digested vesicles. The greater abundance of GGA1 over COPB2 is consistent with previous studies where Rab GTPases involved in anterograde trafficking are more abundant within the *T. gondii* PV than proteins participating in retrograde pathways (6, 15).

Arf1 could also be recruited to supply lipids through either lipid droplet metabolism or non-vesicular trafficking of lipids. Lipid droplets store the precursors of membrane lipids and are released by Arf1 as nano-lipid droplets for further digestion (41). Recent studies have determined that *T. gondii* infection promotes lipid droplet formation and causes them to rearrange around the parasite before their internalization and degradation within the PV (42–44). Furthermore, Arf1 plays a crucial role in activating two proteins involved in non-vesicular lipid transport: ceramide transporter 1 (CERT1), which facilitates the transport of ceramides from the ER to the Golgi, and pleckstrin homology domain-containing A (PLEKHA8), which transports glucosylceramides within the Golgi and from the trans-Golgi to the plasma membrane (45). Finally, Arf1 plays a critical role in forming the ER-Golgi intermediate compartment, an essential process for transporting the major histocompatibility complexes to the cell surface and the regulation of immune mediators’ secretion (46). Thus, disruption of ER-Golgi biogenesis by trafficking Arfs into the PV could impact antigen presentation and immune responses.

We also reported that Arf1 recycling between its GDP/GTP-bound states facilitated its internalization into the *T. gondii* PV and overexpression of either Arf1-Q71L or Arf1-T31N resulted in a significant reduction in PV size at 48 hpi. This phenotype indicates that functional Arf1 protein or Arf1-mediated functions facilitate proper *T. gondii* development. Future studies may resolve if Arf recycling directly impacts *T. gondii* size or if host-dependent functions of Arf are influencing the parasite. However, the finding that Arf1 recycling did not depend on host GEF function hints at a possible parasite protein that can regulate Arf activation. In bacterial systems, secreted effector proteins that mimic Arf GTPase partners can modulate host Arfs. For instance, *L. pneumophila* secretes the bacterial effector protein RalF, which contains a Sec7 domain that activates host Arf1 for recruitment to its vacuole (19). Conversely, enterohemorrhagic *Escherichia coli* secretes a type III effector protein, EspG, that binds GTP-bound Arf1 and inhibits bidirectional Golgi trafficking (47, 48). It is therefore possible that *T. gondii* and *P. berghei* could export proteins into the host cytosol to modulate host protein machinery and recruit specific vesicles to its PV.

Altogether we have demonstrated that host Arfs play a critical role during the *T. gondii* lytic cycle and the *P. berghei* liver stage, where each parasite uniquely exploits the protein family. This work builds on the evidence that Arf and Rab GTPases are hijacked by apicomplexan parasites possibly to exploit vesicular trafficking. Importantly, we provide a comprehensive analysis of Arf1 internalization into the *T. gondii* PV and identify trafficking machinery essential for *P. berghei* liver stage survival. Additional studies are critical to understand how parasites redirect different GTPases, access their cargo, and undergo nutrient exchange. Further exploration into why Arfs are manipulated by apicomplexans will uncover whether they function to support nutrient acquisition and/or prevent elimination. There is also a need to identify exported and PVM-resident proteins for both *T. gondii* and *P. berghei* as a framework to discover candidate protein(s) that may regulate these processes. Continued efforts to resolve the role of Arf GTPases will help lay the groundwork to identify essential and targetable pathways to inhibit apicomplexan parasites for disease control.

## MATERIALS AND METHODS

### Cell and parasite culture

HeLa (Duke Cell Culture Facility) and HuH7 (Sigma, European Collection of Authenticated Cell Cultures) were cultured in DMEM (Gibco) supplemented with 10% heat-inactivated FBS (Sigma) and 1% antibiotic-antimycotic (Sigma). Cells were maintained in a standard tissue culture incubator at 37°C and 5% CO_2_. *T. gondii* RH-88 stably expressing mCherry (*Tg-mCh*), generously gifted by Prof. Laura Knolls (University of Wisconsin Madison), and an untagged *T. gondii* RH-88 (BEI, NR-223) were propagated in HeLa cells by serial passage. Parasites were released from host cells by syringe lysis and passed through a 0.3-µm filter. *Anopheles stephensi* mosquitos infected with *Plasmodium berghei* ANKA (*Pb-*Luc) stably expressing luciferase (Luc) were purchased from the New York University Langone Medical Center Insectary or the SporoCore at the University of Georgia, Athens. *P. berghei* sporozoites were harvested from freshly dissected *A. stephensi* salivary glands before experiments. Following the addition of *T. gondii* and *P. berghei* to mammalian cells, plates were centrifuged at 500 × *g* for 10 minutes at room temperature (RT).

### Transfections and knockdowns

For transfections, 3,000 HeLa cells were seeded on 384-well glass bottom black plates (Cellvis-MSPP-P38415HN). Twenty-four hours post-seeding, plasmids (75 ng/well), see Table S2, were transfected with 0.035 µL Lipofectamine 3000 reagent (Invitrogen) per well according to the manufacturer’s protocol. Protein expression was confirmed by microscopy. Cells recovered for 24 hours before infection with 6,000 *T*. *gondii* tachyzoites or 4,000 *P*. *berghei* sporozoites per well.

For knockdowns, 2,000 HuH7 cells were reverse-transfected with Lipofectamine RNAiMax (Invitrogen) in 384-well plates (Corning #3570) with 15 nM final concentration of pooled (2/gene) or single siRNAs against a non-targeting scramble control, *Arf1*, *Arf4*, or *GBF1* (Qiagen). Cells were incubated for 48 hours and subsequently infected with 4,000 *Pb*-Luc sporozoites per well.

### GEF inhibitor studies

To evaluate the role of host GEF activity on Arf1 recruitment, 3,000 HeLa cells were seeded on 384-well glass bottom plates, transfected with Arf1-HA plasmids, and infected with *Tg-mCh* as described above. At 21 or 29 hpi, infected cells were treated with 3.5 µM golgicide A (APExBIO; Cat# B1385), 1 µM brefeldin A (APExBIO; Cat# B1400), or 1% DMSO (vehicle). Cells were fixed as described below after 3 hours of treatment.

### Sphingolipid acquisition studies

HeLa cells were seeded on 384-well glass bottom plates, transfected with Arf1-GFP, and infected with an untagged *T. gondii* RH-88 strain as described above. At 24 hpi, cells were incubated with 5 µM BODIPY TR C5-ceramide complexed to BSA (Invitrogen; Cat# B34400) in FBS-free DMEM. Nuclei were stained with Hoechst (1:2,000 in PBS) for 5 minutes before live cell microscopy at 48 hpi.

### RNA extraction and qRT-PCR

*Arf1* mRNA levels during *T. gondii* infection were assessed by qRT-PCR. HeLa cells seeded in 6-well plates (300,000 cells/well) were infected with 1.5 million *T*. *gondii* tachyzoites. Uninfected and *T. gondii-*infected cells were harvested at 4, 24, and 48 hpi with RNA lysis buffer (Zymo Research). RNA was harvested per the manufacturer’s protocol using the Quick-RNA MiniPrep kit (Zymo Research). First-strand cDNA synthesis was performed with 0.5 µg of RNA using random hexamers (Invitrogen) and the GoScript reverse transcriptase (Promega) per the manufacturer’s instructions. qRT-PCR analysis was performed with oligonucleotide primers in Table S3 with 2× universal SYBR green fast qPCR (Abclonal) and a LightCycler 480 Instrument II (Roche Diagnostics). All reactions were at a final volume of 5 µL in a 384-well plate (Roche 04729749001). Cycle threshold (CT) values for the genes were normalized to CT values of the 18S human housekeeping gene rRNA (CT[target] -CT[18S rRNA] = ∆CT). Data were normalized to uninfected cells (∆CT[experimental] − ∆CT[control] = ∆∆CT) and the relative amount was calculated as 2^−∆∆CT^.

The mRNA levels of genes targeted by siRNA were measured 48 hours post-reverse transfection. Six pooled wells of a 384-well plate were used to extract total RNA with the Quick-RNA MicroPrep per the manufacturer’s instructions (Zymo Research). First-strand cDNA synthesis and qRT-PCR were performed as described above.

### Protein expression and western blot

*T. gondii* RNA extraction and first-strand cDNA synthesis were performed as described above. *TgArf1* was PCR amplified from cDNA using the primers in Table S2 for cloning into the pET21a(+) expression vector in frame with a C-terminal His-tag. Plasmids were verified by Sanger Sequencing (Eton Bioscience) and transformed into BL21(DE3) *E. coli*. Protein expression was induced at OD_600_ = 0.6 with 100 µM IPTG (Chem Impex, cat #00194) overnight at 20°C. One milliliter of culture was pelleted and resuspended in 200 µL of denaturing lysis buffer (0.1 M Tris-HCl, pH 8, 0.1 M Na_2_PO_4_, 8 M urea). For HeLa lysate, confluent cells in a T75 flask were trypsinized, washed three times with PBS, and resuspended in 200 µL of denaturing lysis buffer. Both BL21-*Tg*Arf1 and HeLa lysates were cleared by centrifugation at 20,000 *× g* for 20 minutes, boiled, and resolved on a Novex 4-20% tris-glycine gel. Proteins were transferred to a nitrocellulose membrane using a Trans-Blot Turbo transfer system (Bio-Rad), blocked for 1 hour with 3% BSA in PBS containing 0.2% Tween 20 (PBST), and incubated with the following primary antibodies overnight at 4°C: Arf1 polyclonal antibody (Invitrogen PA1-127, 1:1000) and Arf1 monoclonal antibody (ThermoScientific 1862342, 1:1000). The membranes were washed three times with PBST before incubation with Invitrogen Alexa Fluor conjugated antibodies (A11001 and A21206, 1:1000). Membranes were washed three times before imaging with a ChemiDoc system.

### *P. berghei* luciferase assay and cell viability

Forty-eight hours post-infection with *Pb-*Luc*,* hepatocytes were assessed for cell viability using CellTiter-Fluor (Promega). The relative fluorescence signal intensity was evaluated with an EnVision plate reader (PerkinElmer). Parasite load from the same microplate was then assessed using Bright-Glo (Promega). Immediately after reagent addition, the relative bioluminescence signal intensity was evaluated. Data were normalized to negative controls.

### Immunofluorescence microscopy

*T. gondii*- and *P. berghei-*infected cells were fixed at 4, 24, 32, or 48 hpi with 4% paraformaldehyde (Sigma) in PBS for 15 minutes at either 37°C or RT. Fixed cells were washed three times with PBS, permeabilized with 0.1% Triton X-100 (Fisher Scientific) for 10 minutes at RT, washed three times again with PBS, and then blocked with 3% BSA and 0.05% Tween-20 (Millipore Sigma) in PBS (blocking buffer) for 1 hour at RT. Cells were subsequently stained with a primary antibody diluted in blocking buffer for 1 hour at RT or overnight at 4°C. Primary antibodies include mouse monoclonal anti-HA (Santa Cruz Cat# sc-7392, 1:400), rabbit monoclonal anti-HA (Cell Signaling Technology Cat# 3724, 1:400), rabbit monoclonal anti-GM130 (Abcam Cat# 52649, 1:200), rabbit monoclonal anti-GBF1 (Abcam Cat# 189512, 1:300), rabbit monoclonal anti-Arf4 (Abcam Cat# AB171746, 1:100), mouse monoclonal anti-V5 (Invitrogen Cat# R96025, 1:700), goat polyclonal anti-UIS4 (Antibodies.com Cat# A121573, 1:1000), and phalloidin-iFluor 647 (abclonal Cat# ab176759). Following three washes with PBS, cells were incubated with Invitrogen Alexa Fluor-conjugated secondary antibodies in blocking buffer (1:400) for 1 hour at RT (A11001, A11005, A21206, A10037, A10042, and A31573). Cells were washed three times, incubated with Hoechst (1:50,000 in PBS) for 10 minutes at RT, and then washed an additional three times with PBS. Fixed cells were viewed on a Zeiss Airyscan 880 inverted confocal microscope with a Märzhäuser linearly encoded x,y stage and a 63 × 1.4 NA oil-immersion plan Apochromat objective. Laser illumination was *via* Argon for 488 nm, diode for 405 nm and 561 nm, and HeNe for 633 nm. The fluorescence signal was collected with two photomultiplier tubes and one GaAsP detector in the following emission ranges: for DAPI—415–487nm, for Alexa Fluor 488–490–570 nm GaAsP, for BODIPY-TR, mCherry, and Alexa Fluor 568–570–633 nm (GaAsP), and Alexa Fluor 647–633–670 nm. Images were acquired sequentially by line scanning bidirectionally at 0.52 microseconds per pixel with line averaging of 4 and a size of 0.044 μm × 0.044 µm with pinhole calculated to be 1 airy unit for green or far-red emission using Zeiss Zen software (version 2.3) and saved as Carl Zeiss Image files. Z-stacks were acquired with 410 nm intervals with approximately 15–25 slices per stack.

### Image and data analysis

Image analysis to adjust brightness, contrast, and crop images was conducted with FIJI (Schindelin *et al*. 2012). Positive internalization of Arf proteins was assessed on the middle focal plane of the vacuole and was considered positive if the vacuole contained at least one punctum. The mean fluorescent intensity values within the PV were assessed on one focal plane using images acquired with the same laser intensity and exposure time for all channels. For internalization, mean fluorescent intensity, and MCC of the *T. gondii* vacuole area was defined by the outermost perimeter of the *Tg-mCh* rosette.

Imaris (version 9.9.1) was used to study the accumulation of Arf4 and GBF1 in the parasite vacuole. A surface of the PVM was generated by the UIS4 signal and Arf4 and GBF1 spots within the host cell boundary, determined by phalloidin staining, were identified. Positive attraction was assessed using the cumulative count plot where Arf4 or GBF1 spots deviated from 98% of simulations (1,000) for the random distribution of spots. To assess the average distance around the parasite vacuole where Arf4 and GBF1 accumulated, we used the smoothed probability density plot where the accumulation of spots around the surface was greater than the random distribution.

All experiments were completed in triplicate with more than 50 PVs analyzed per condition, except where noted. Data were analyzed using GraphPad Prism10.

## SUPPLEMENTAL MATERIAL

Fig. S1 msphere.00770-23-s0001.tiff Western blot probing Arf1.

Fig.S2 msphere.00770-23-s0002.tiff Host Arf recruitment during *T. gondii* infection.

Fig. S3 msphere.00770-23-s0003.tiff Internalization of Arf1 does not depend on host expression.

Fig. S4 msphere.00770-23-s0004.tiff Arf1 expression by qRT-PCR.

Fig. S5 msphere.00770-23-s0005.tiff Arf1-WT and mutant Arf1 are not internalized into all *T. gondii* PVs.

Fig. S6 msphere.00770-23-s0006.tiff YFP-GBF1 internalization into *T. gondii* PV.

Fig. S7 msphere.00770-23-s0007.tiff Host GEF function is not required at 32 hpi.

Fig. S8 msphere.00770-23-s0008.tiff Manders' colocalization coefficient of GFP and Arf-HA.

Fig. S9 msphere.00770-23-s0009.tiff Examples of negative Arf4 and GBF1 recruitment in *P. berghei*-infected hepatocytes.

Supplemental legends and tables msphere.00770-23-s0010.docx Legends for supplemental figures; Tables S1 to S3.

ASM does not own the copyrights to Supplemental Material that may be linked to, or accessed through, an article. The authors have granted ASM a non-exclusive, world-wide license to publish the Supplemental Material files. Please contact the corresponding author directly for reuse.
