# Supplementary figures and images for: Toxoplasma and Plasmodium associate with host Arfs during infection
Source: mSphere. 2024 Feb 13;9(3):e00770-23. doi: 10.1128/msphere.00770-23 (PMC10964417; doi:10.1128/msphere.00770-23)

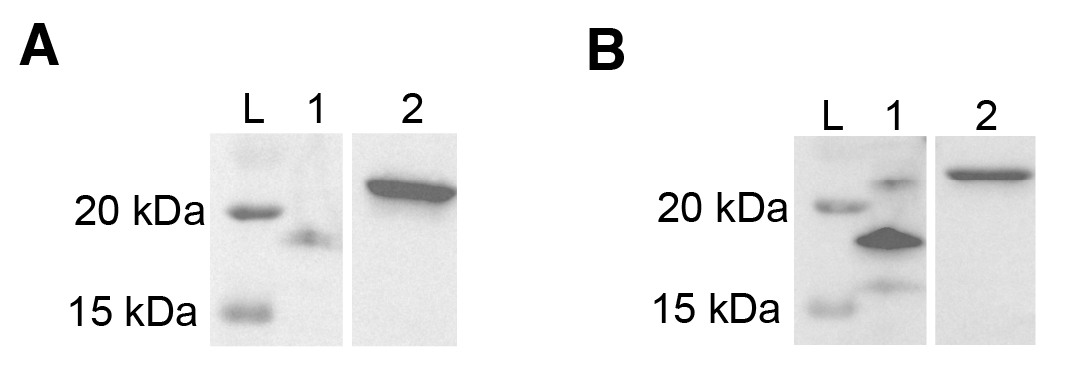

Supplement: Fig. S1 — Western blot probing Arf1. [file msphere.00770-23-s0001.tiff]

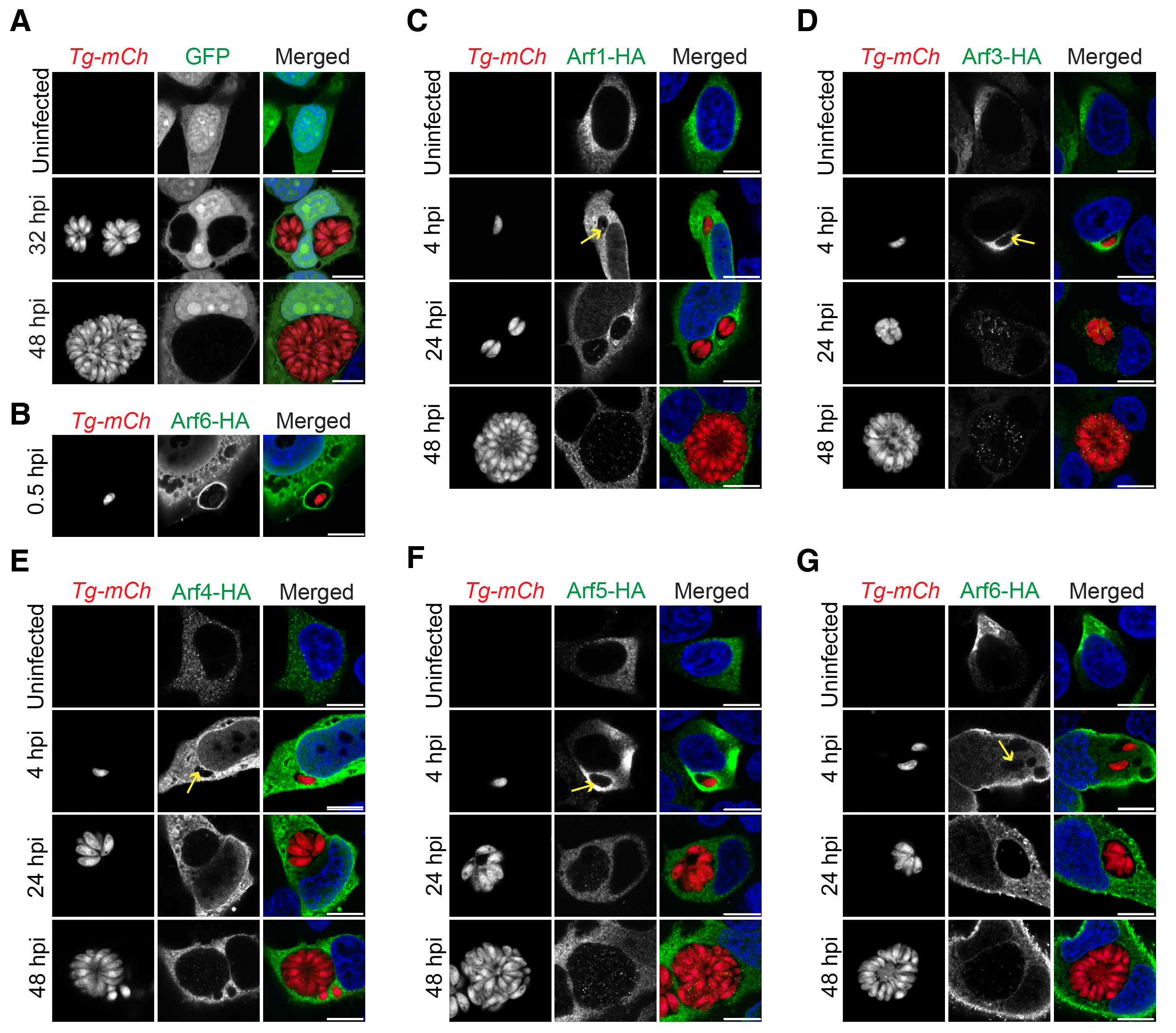

Supplement: Fig.S2 — Host Arf recruitment during T. gondii infection. [file msphere.00770-23-s0002.tiff]

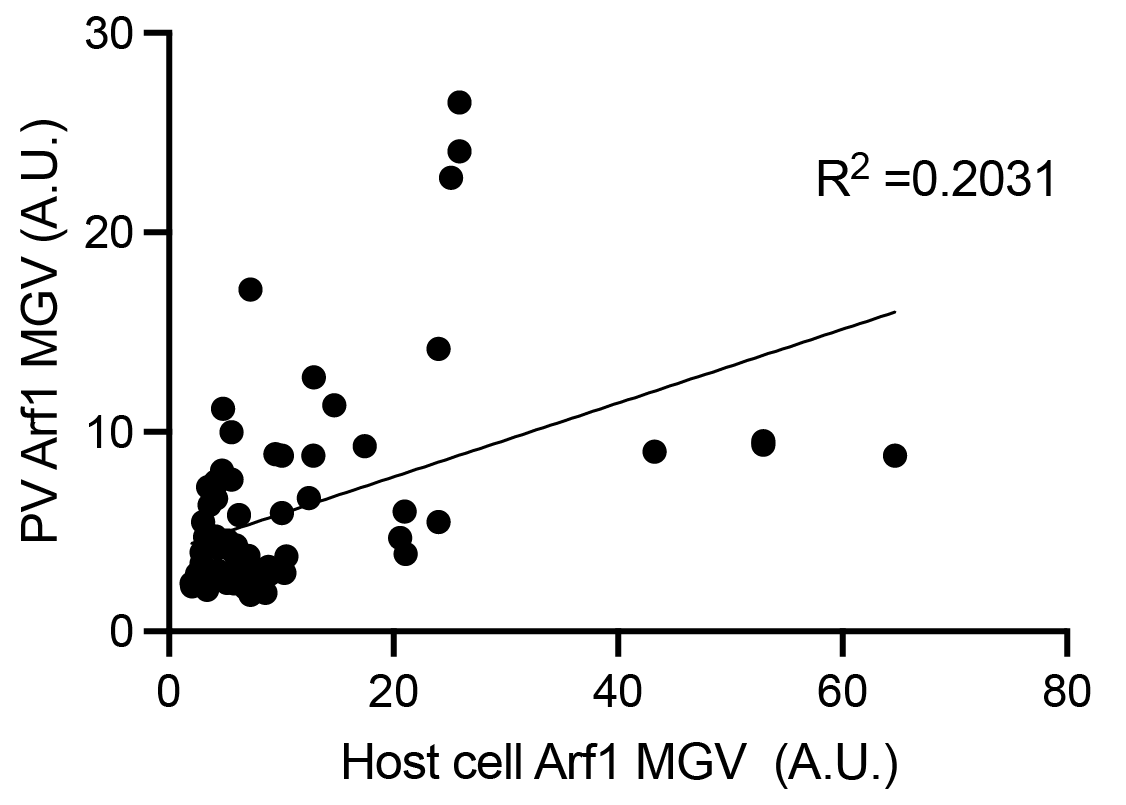

Supplement: Fig. S3 — Internalization of Arf1 does not depend on host expression. [file msphere.00770-23-s0003.tiff]

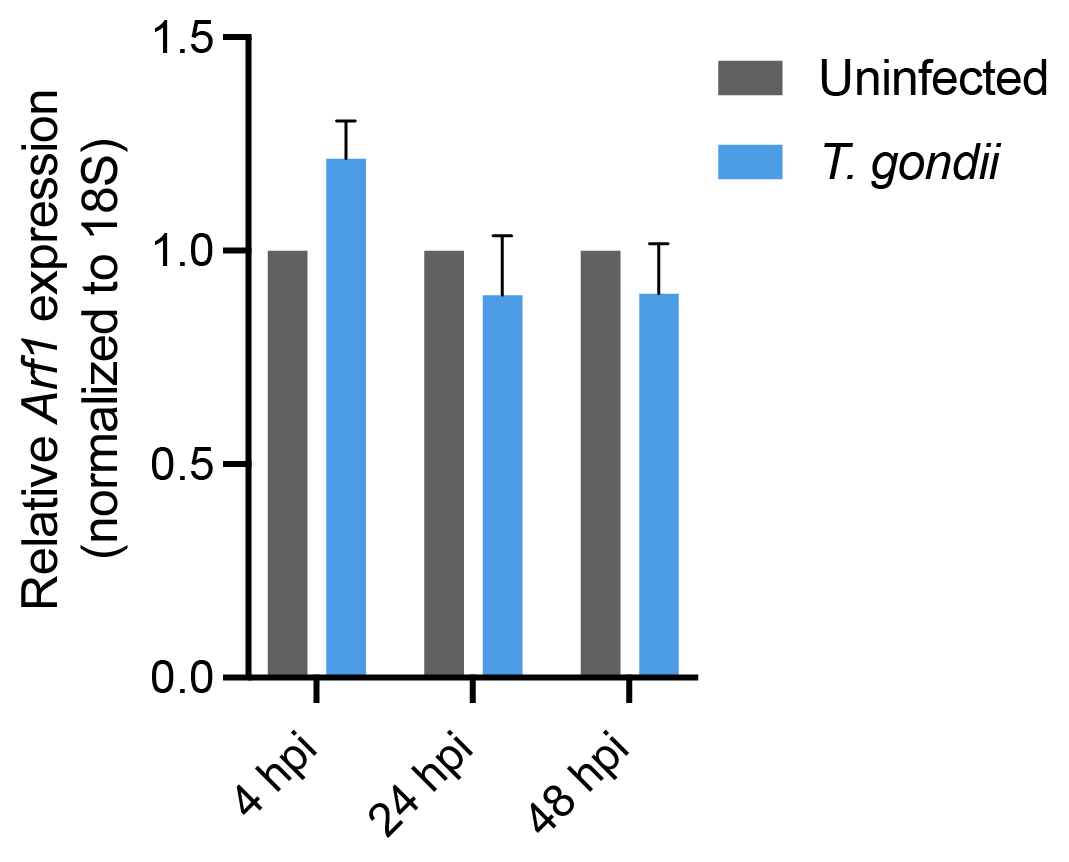

Supplement: Fig. S4 — Arf1 expression by qRT-PCR. [file msphere.00770-23-s0004.tiff]

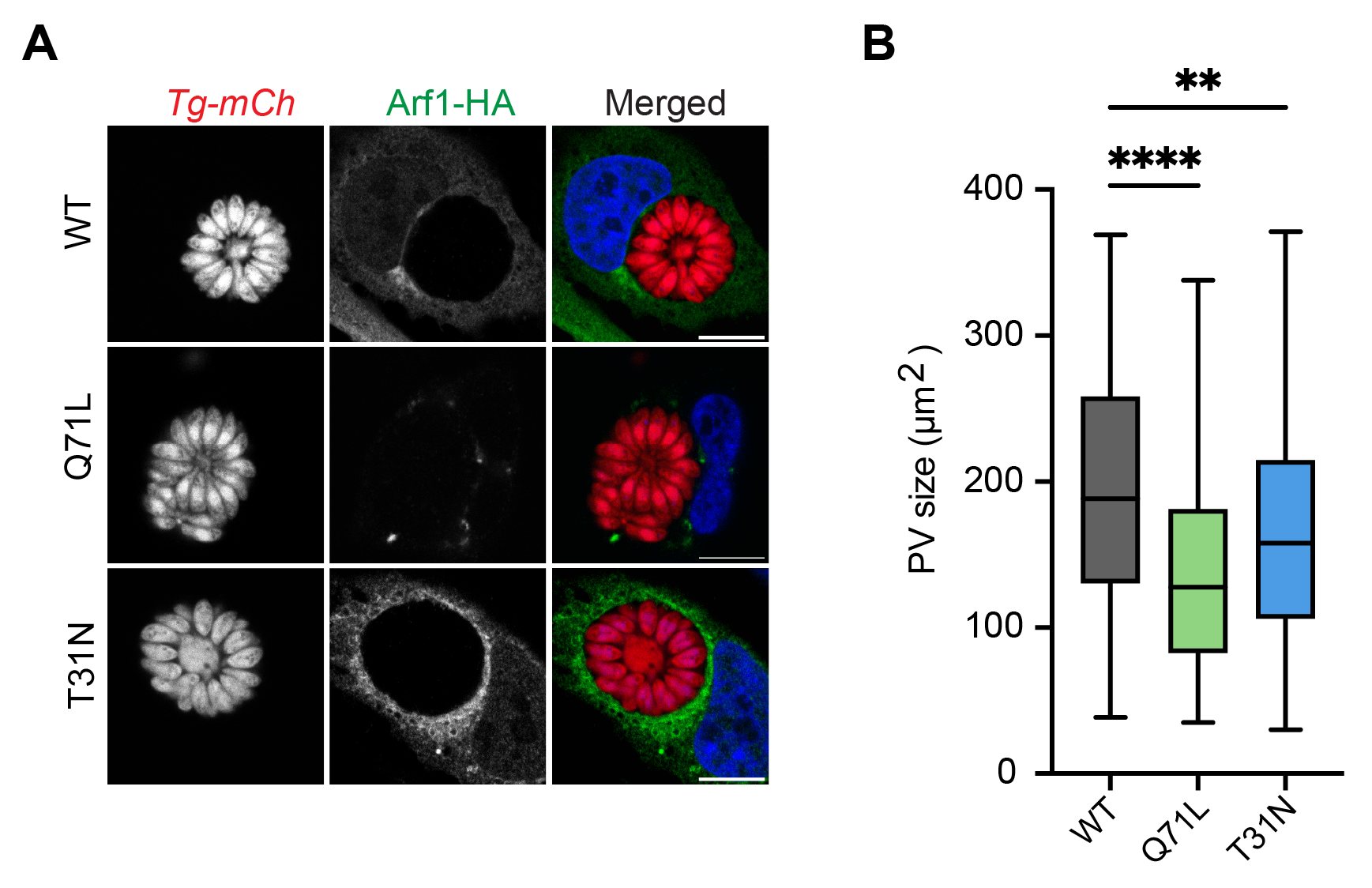

Supplement: Fig. S5 — Arf1-WT and mutant Arf1 are not internalized into all T. gondii PVs. [file msphere.00770-23-s0005.tiff]

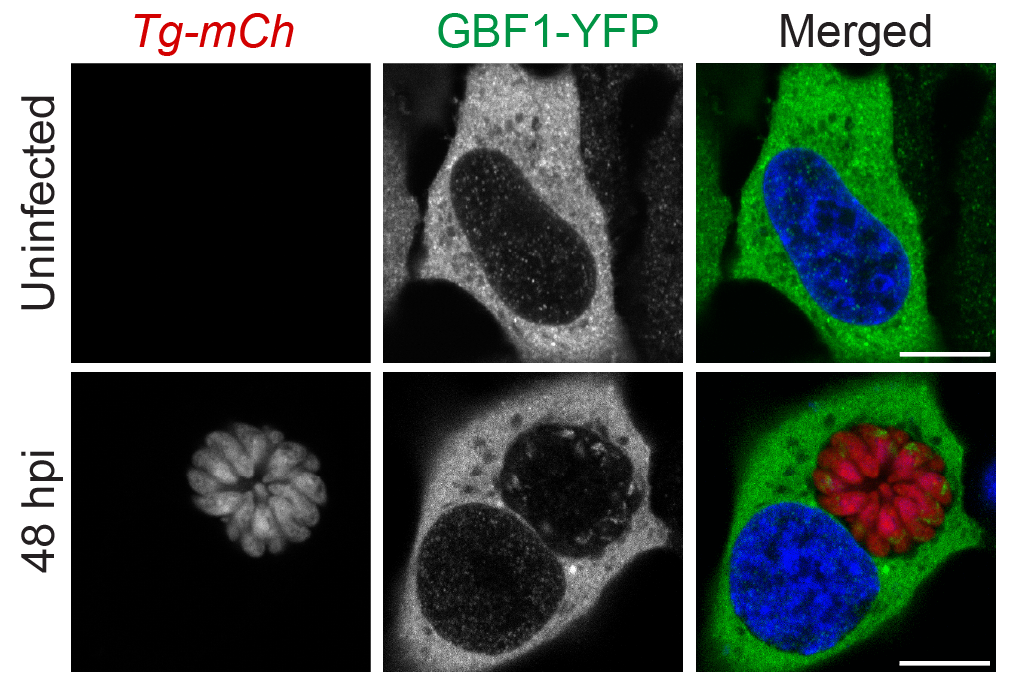

Supplement: Fig. S6 — YFP-GBF1 internalization into T. gondii PV. [file msphere.00770-23-s0006.tiff]

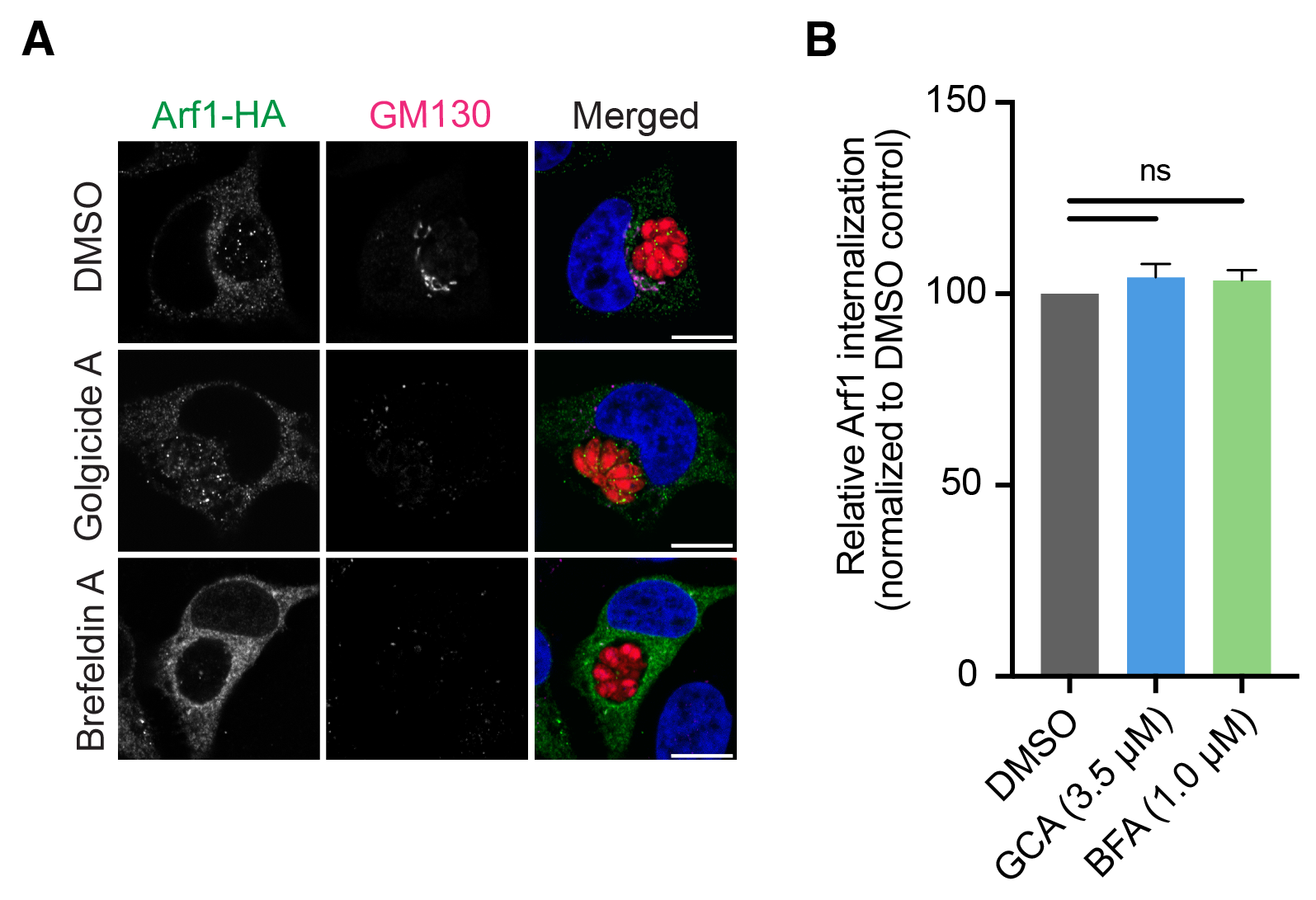

Supplement: Fig. S7 — Host GEF function is not required at 32 hpi. [file msphere.00770-23-s0007.tiff]

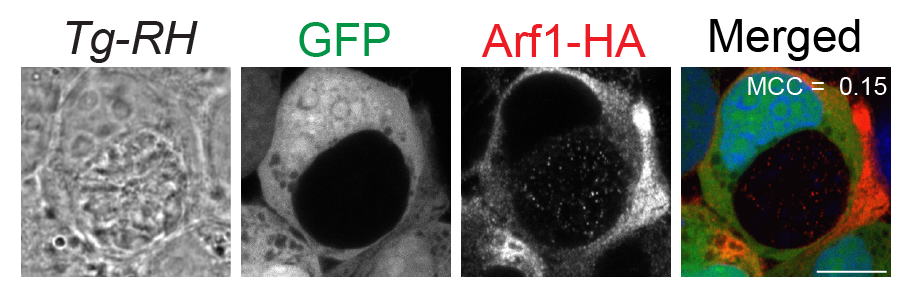

Supplement: Fig. S8 — Manders' colocalization coefficient of GFP and Arf-HA. [file msphere.00770-23-s0008.tiff]

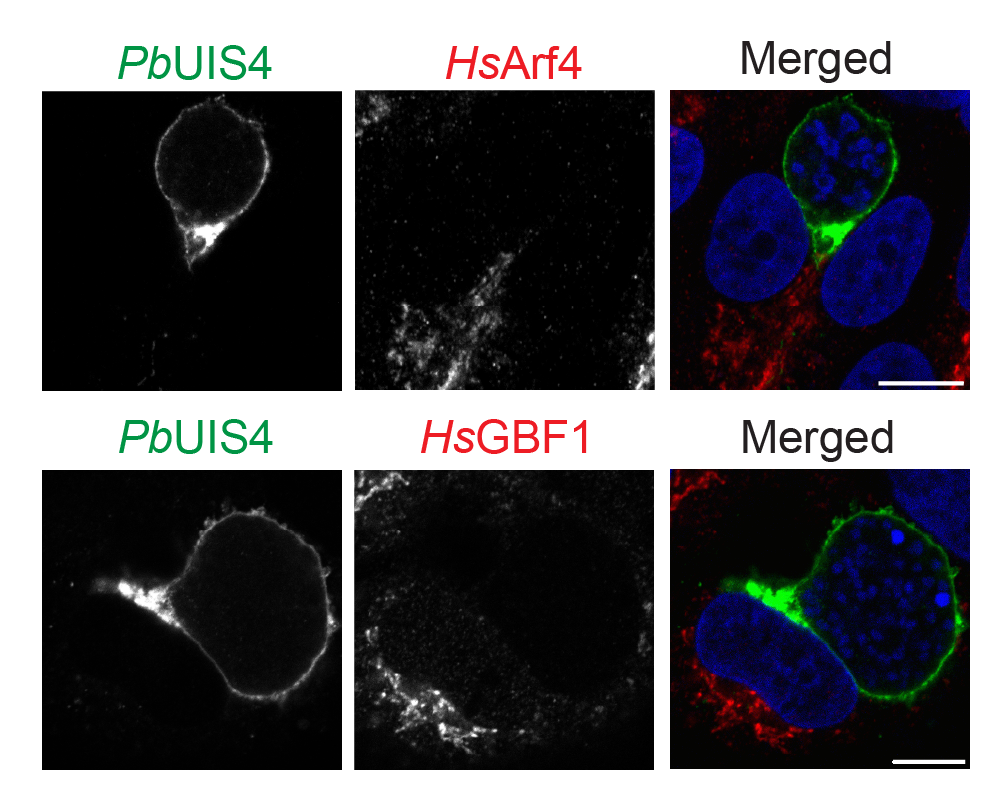

Supplement: Fig. S9 — Examples of negative Arf4 and GBF1 recruitment in P. berghei-infected hepatocytes. [file msphere.00770-23-s0009.tiff]
