# Supplementary material for: Toxoplasma and Plasmodium associate with host Arfs during infection
Source: mSphere. 2024 Feb 13;9(3):e00770-23. doi: 10.1128/msphere.00770-23 (PMC10964417; doi:10.1128/msphere.00770-23)
Supplement: Supplemental legends and tables — Legends for supplemental figures; Tables S1 to S3. [file msphere.00770-23-s0010.docx]

**Supplemental Figure 1: Arf1 antibody cross-reaction with *Tg*Arf1.** Western blot probing for human Arf1 (21 kDa, observed at ~18 kDa consistent with previous reports) in HeLa cell lysate and *T. gondii* Arf1-His_6_ (22.1 kDa) in *E. coli* lysate using an **(A)** Arf1 polyclonal antibody from Invitrogen (PA1-127) and **(B)** an Arf1 monoclonal antibody from ThermoScientific (1862342). Lanes: (L) Bio-Rad Precision Plus Protein Dual Color Standards; (1) HeLa lysate; and (2) *E. coli* lysate overexpressing *Tg*Arf1-His_6_.

**Supplemental Figure 2: Host Arf recruitment during *T. gondii* infection of HeLa cells. (A)** Representative confocal immunofluorescence microscopy images of uninfected and Tg-mCh (red)-infected HeLa cells overexpressing GFP (green) at 32 and 48 hpi. GFP was not observed in the *T. gondii* PV. Nuclei were stained with Hoechst (blue). **(B)** Tg-mCh (red)-infected HeLa cells overexpressing Arf6-HA at 0.5 hpi. A gathering of Arf6-HA is observed during parasite invasion. (**C-G**) Grayscale images from Figure 1. Representative confocal immunofluorescence microscopy images of uninfected and Tg-mCh (red)-infected HeLa cells overexpressing (**C**) Arf1, (**D**) Arf3, (**E**) Arf4, (F) Arf5, and (**G**) Arf6. Nuclei are stained with Hoechst (blue). Scale bars are 10 μm.

**Supplemental Figure 3: Internalization of Arf1 does not depend on host expression.** Plot showing the mean Arf1 fluorescent intensity values within the host cell compared to the fluorescent intensity within the *T. gondii* PV of infected cells at 48 hpi. n = 3 biological replicates analyzing a minimum of 20 PVs per replicate. A linear regression analysis is shown. Data do not fit a linear equation suggesting that observed Arf PV internalization is not a function of the level of protein expression by the host cell.

**Supplemental Figure 4: *Arf1* expression remains constant during *T. gondii* lytic cycle.** Relative *Arf1* mRNA levels of uninfected and *T. gondii-*infected HeLa cells at 4, 24, and 48 hpi measured by qPCR. *Arf1* was normalized to *Hs*18S at each timepoint. Data represent mean ± SEM, n = 3 biological replicates.

**Supplemental Figure 5: Arf1-WT and mutant Arf1 are not internalized into all *T. gondii* PVs. (A)** Representative confocal immunofluorescence images of Tg-mCh (red)-infected HeLa cells overexpressing Arf1-WT, the constitutively active mutant Arf1-Q71L, and the dominant-negative mutant Arf1-T31N (green) at 48 hpi. Nuclei were stained with Hoechst (blue). Images are examples of *T. gondii* PVs scored as negative for internalization. Scale bars are 10 μm. **(B)** Box and whisker plot showing the *T. gondii* PV size (μm^2^) for Tg-mCh-infected HeLa cells overexpressing Arf1-WT (gray), Arf1-Q71L (green), and Arf1-T31N (blue) at 48 hpi. n = 3 biological replicates analyzing a minimum of 50 PVs per condition. *P*-values display one-way ANOVA with Dunnett’s multiple comparison test for each condition compared to Arf1-WT. ***P* <0.01; *****P* <0.0001.

**Supplemental Figure 6: YFP-GBF1 internalized into *T. gondii* PV.** Representative confocal immunofluorescence images of uninfected and Tg-mCh (red)-infected HeLa cells overexpressing YFP-GBF1 (green) at 48 hpi. Nuclei are stained with Hoechst (blue). Scale bars are 10 μm.

**Supplemental Figure 7: Host GEF function is not required for Arf1 internalization at 32 hpi. (A)** Representative confocal immunofluorescence images of Tg-mCh (red)-infected HeLa cells overexpressing Arf1-HA and treated with DMSO, 3.5 μM Golgicide A (GCA), or 1.0 μM Brefeldin A (BFA). Drug treatment was from 29-32 hpi before cells were fixed at 32 hpi. Cells were stained with anti-HA (green), anti-GM130 (magenta), and nuclei were stained with Hoechst (blue). **(B)** Quantification of *T. gondii* PVs with at least one punctum of Arf1 at 32 hpi after treatment with DMSO (gray), GCA (blue), or BFA (green). Data represents the relative recruitment of Arf1 compared to the DMSO control ± SEM, n = 3-4 biological replicates analyzing > 50 PVs for each condition. *P*-values display one-way ANOVA with Dunnett’s multiple comparison test for each condition compared to DMSO. ns = non-significant. Scale bars are 10 μm.

**Supplemental Figure 8: Manders’ colocalization coefficient of GFP and Arf1-HA co-transfected cells.** Representative confocal immunofluorescence images of *Tg-RH*-infected HeLa cells overexpressing GFP (green) and Arf1-HA (red) at 48 hpi. Nuclei were stained with Hoechst (blue). The Manders’ colocalization coefficient (MCC) inside of the PV was determined for GFP to Arf1. Scale bars are 10 μm.

**Supplementary Figure 9: Examples of negative Arf4 and GBF1 recruitment in *P. berghei-*infected hepatocytes.** Representative confocal immunofluorescence images of *P. berghei* (green)*-*infected HuH7 cells at 48 hpi. Cells are stained with anti-UIS4 (green), anti-Arf4 (red) or anti-GBF1 (red), and nuclei are stained with Hoechst (blue). Images are examples of *P. berghei* PVs scored as negative for accumulation of Arf4 and GBF1. Scale bars are 10 μm.

**Supplementary Table 1: RNA-seq studies of host Arf expression during *T. gondii* infection.**

|  | **Pittman *et al.* 2014^*#^** (Acute infection, 10 dpi) | **Pittman *et al.* 2014^*#^**  (Chronic infection, 28 dpi) | **Ulahannan *et al.* 2022^#^**  (Human foreskin fibroblast, 24 hpi) |
| --- | --- | --- | --- |
| ***Arf1*** | -0.145 (0.205) | 0.073 (0.464) | -0.140 (0.409) |
| ***Arf3*** | -0.133 (0.252) | -0.127 (0.252) | -0.122 (0.436) |
| ***Arf4*** | 0.223 (0.180) | 0.581 (5e-05) | -0.174 (0.312) |
| ***Arf5*** | -0.351 (0.015) | -0.027 (0.825) | -0.107 (0.535) |
| ***Arf6*** | 0.765 (0.238) | 0.975 (5e-05) | 0.042 (0.799) |

* Samples were collected from the forebrains of mice during acute infection when tachyzoites are the prominent parasite form and during chronic infection when bradyzoite cysts have developed.

# Values represent Log2(Fold_Change) (p-value)

**Supplementary Table 2: Plasmids used in the study.**

| **Name** | **Source** | **Citation** |
| --- | --- | --- |
| pcDNA3/hArf1(WT)-HA | Addgene 79409 | Kondo *et al.* 2012 |
| pEGFP-N1/Arf1-GFP | Addgene 39554 | Chun et al. 2008 |
| pcDNA3 HA Arf1 ActQ71L | Addgene 10832 | Furman *et al.* 2002 |
| pcDNA3 HA Arf1 DN T31N | Addgene 10833 | Furman *et al.* 2002 |
| pcDNA3/hArf3(WT)-HA | Addgene 79414 | Kondo *et al.* 2012 |
| pCAG/hArf4(WT)-HA | Addgene 79403 | Nakai *et al.* 2013 |
| pcDNA3/Arf4(WT)-HA | This study |  |
| pcDNA3/hArf5(WT)-HA | Addgene 79427 | Hanai *et al.* 2016 |
| pcDNA3/hArf6(WT)-HA | Addgene 79424 | Makyio *et al.* 2012 |
| GBF1-YFP | Gift of Prof. Cathy Jackson and Jean-Marc Verbavatz, Institut Jacques Monod | Niu *et al.* 2005 |
| pcDNA3/GFP | This study |  |
| PET-21a(+) *Tg*Arf1-His | This study |  |
| pEGFP-C1/GGA1-GFP | Addgene 178459 | Puertollano *et al.* 2003 |
| pcDNA3/GGA1-V5-His | This study |  |
| pcDNA3/COPB2-V5-His | This study |  |

**Supplementary Table 3: Primers used in the study.**

| **Target** |  | **Sequence (5’ 🡪 3’)** |
| --- | --- | --- |
| Arf1 | Forward | GACCACCATTCCCACCATAG |
|  | Reverse | AACACCAGGAGGACAGCATC |
| Arf4 | Forward | GGGATGTTGGTGGTCAAGAT |
|  | Reverse | AGCAGCACTGCATCTCTCAA |
| GBF1 | Forward | CTCCCAGAAAGAAGGCACAG |
|  | Reverse | GCTACAGGGGCTGACTCAAG |
| 18S rRNA | Forward | GGCCCTGTAATTGGAATGAGTC |
|  | Reverse | CCAAGATCCAACTACGAGCTT |
| *Tg*Arf1 | Forward | AAAAAAGCGGCCGCATGGGTTTGAGCGTCAGCC |
|  | Reverse | AAAAAACTCGAGATCGATGTTTTTCTGCGCA |
